# Conducting Polymer-Reinforced Laser-Irradiated Graphene as a Heterostructured 3D Transducer for Flexible Skin Patch Biosensors

**DOI:** 10.1021/acsami.1c13164

**Published:** 2021-11-02

**Authors:** Lingyin Meng, Anthony P. F. Turner, Wing Cheung Mak

**Affiliations:** Biosensors and Bioelectronics Centre, Division of Sensor and Actuator Systems, Department of Physics, Chemistry and Bi-ology, Linköping University, SE-581 83 Linköping, Sweden

**Keywords:** laser-irradiated graphene, conducting polymers, heterostructured 3D transducers, skin patch, wearable biosensors

## Abstract

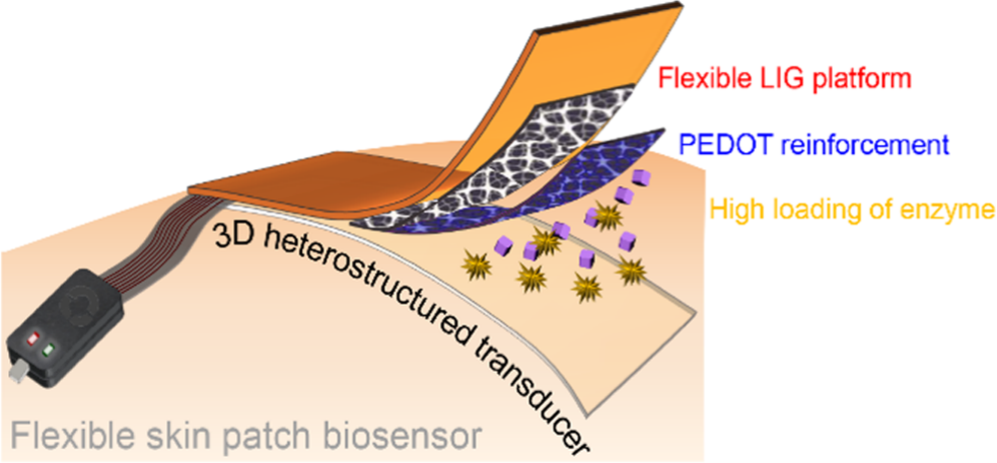

Flexible skin patch biosensors are promising for the noninvasive determination of physiological parameters in perspiration for fitness and health monitoring. However, various prerequisites need to be met for the development of such biosensors, including the creation of a flexible conductive platform, bending/contact stability, fast electrochemical kinetics, and immobilization of biomolecules. Here, we describe a conducting polymer-reinforced laser-irradiated graphene (LIG) network as a heterostructured three-dimensional (3D) transducer for flexible skin patch biosensors. LIG with a hierarchically interconnected graphene structure is geometrically patterned on polyimide via localized laser irradiation as a flexible conductive platform, which is then reinforced by poly(3,4-ethylenedioxythiophene) (PEDOT) as a conductive binder (PEDOT/LIG) with improved structural/contact stability and electrochemical kinetics. The interconnected pores of the reinforced PEDOT/LIG function as a 3D host matrix for high loading of “artificial” (Prussian blue, PB) and natural enzymes (lactate oxidase, LOx), forming a compact and heterostructured 3D transducer (LOx/PB-PEDOT/LIG) for lactate biosensing with excellent sensitivity (11.83 μA mM^–1^). We demonstrated the fabrication of flexible skin patch biosensors comprising a custom-built integrated three-electrode system achieve amperometric detection of lactate in artificial sweat over a wide physiological linear range of 0–18 mM. The advantage of this facile and versatile transducer is further illustrated by the development of a folded 3D wristband lactate biosensor and a dual channel biosensors for simultaneous monitoring of lactate and glucose. This innovative design concept of a heterostructured transducer for flexible biosensors combined with a versatile fabrication approach could potentially drive the development of new wearable and skin-mountable biosensors for monitoring various physiological parameters in biofluids for noninvasive fitness and health management.

## Introduction

The current trend in diagnostics is moving from conventional invasive point-of-care tests toward noninvasive flexible and wearable devices for continuous, real-time, and remote monitoring of an individual’s physiological conditions and combining this with mobile technologies to deliver healthcare in decentralized locations in a far more cost-effective manner. Over the past decade, various ingenious electrode configurations have been exploited as flexible and wearable electrochemical sensors and biosensors to measure an individual’s physical parameters, such as skin temperature, body motion, heart rate, and respiration rate,^1−3^ and physiological metabolites in sweat, saliva, or tears, such as glucose, lactate, alcohol, hormones, and electrolyte ions.^4−6^ The fabrication of these platforms usually involves a complicated procedure including mask-patterning and deposition of conductive electrodes and consequent functionalization on a flexible substrate for signal transduction.^4,5,7^

Recently, laser irradiation technology has been employed for contact-free and mask-free construction of conductive carbon patterns from thermoset polyimide or other precursors (*e.g.*, graphene oxide, wood, and paper).^8−13^ Under laser irradiation, the polymer precursors undergo dissociation due to a photothermal/photochemical effect with the rapid evolution and release of gaseous products (*e.g.*, CO, CO_2_, H_2_O, C_2_H_2_) throughout the film, resulting in porous structures. Simultaneously, the aromatic rings in the precursor molecules reorganize into a graphene structure by the carbonization/graphitization process, yielding a patternable and porous conducting laser-irradiated graphene (LIG).^8,10^ Based on the facile nature of the laser irradiation technology compared to conventional printing processes as well as the resultant porous structure with high conductivity, LIG has been applied for several applications including energy storage,^10,14^ electrocatalysis,^15^ physical sensors,^16,17^ chemical sensors, and biosensors.^18−21^ In addition, the patterning of LIG layered on top of a flexible substrate is also a promising candidate for the construction of flexible and wearable electronic devices, such as face masks,^22^ soft actuators,^23^ and electronic skins.^17,24^ However, LIG with a porous structure is fragile in nature and incompatible with a flexible substrate, which limits its mechanical strength, durability, and stability when subjected to large deformation (bending, twisting, and stretching),^25,26^ and as a result, affects its electrochemical performance when applied in flexible and wearable devices.

Conducting polymers (CPs), as an emerging class of functional π-conjugated organic polymers, offer great potential for use in next-generation electronic devices due to their unique properties of redox reversibility and electronic/ionic conductivity. The improved performance of CPs in electronics, such as facilitated kinetics and electron transfer, relies on CP nanostructures created via various synthetic methods including both template-orientated polymerization (*e.g.*, using hard or soft templates) and physical approaches (*e.g.*, electrospinning and imprinting). Such nanostructured CPs feature new properties including large active surface areas and porous structures while inheriting prominent physical–chemical properties from their bulk polymer equivalents.^27^ On the other hand, CPs are soft with good mechanical flexibility and stability, which make them promising candidates as conductive polymeric binders instead of the commonly used insulating varieties, such as polyacrylic acid and poly(vinyl alcohol). The CPs would serve as a matrix to stabilize the morphology via adhesion and binding for a large variety of advanced materials including inorganic nanoparticles,^28^ metal oxides,^29^ carbon nanotubes,^30^ and graphene,^31^ resulting in improved mechanical properties and structural integrity. In addition, the introduction of CPs as conductive additives can facilitate electrode kinetics and thus enhance the electrochemical performance, such as improved electrochemical sensing of dopamine at PEDOT-modified laser-scribed graphene^32^ and increased capacitive behavior of PEDOT-coated LIG supercapacitors.^33^

More importantly, flexible and wearable sensors for biomarkers necessitate the immobilization of a biomolecular recognition element (*e.g.*, enzyme) as the outermost layer on top of a transducer. Conventional layer-by-layer casting techniques, especially on two-dimensional (2D) planar electrodes, not only limit enzyme loading and distribution but also hinder the mass transport and charge transfer at the enzyme–electrode interface.^34,35^ Such 2D planar enzymatic electrodes are not ideal for flexible and wearable biosensors because of the potential intimate contact of the outermost enzyme layer with a tissue as well as the delamination of the enzyme layer caused by the large deformation (bending, twisting, and stretching) associated with flexible devices, leading to reduced transduction efficiency of enzymatic intermediates with resulting low sensitivity.^7^ Instead of a planar geometry, the construction of a heterostructured three-dimensional (3D) transducer with high porosity is promising to provide a large and accessible surface area, enabling the high loading of an enzyme and its efficient communication with the electrode.^34^ In addition, a compact and heterostructured 3D transducer would provide a friendly host matrix for enzyme immobilization with good long-term operational stability and structural stability.^36^ In the light of this, the facile construction of a conductive network with an *in situ* 3D porous structure for stable and effective immobilization of biomolecules is highly desirable for the preparation of heterostructured 3D transducers for flexible and wearable biosensors with high sensitivity and stability.

Herein, we demonstrate the development of a heterostructured 3D transducer for a flexible skin patch-based biosensor. We optimized the LIG process for the facile and mask-less fabrication of a geometrically patterned 3D porous LIG flexible electrode, followed by reinforcing the hierarchically interconnected graphene structure within the LIG *via* nanodeposition of conducting polymer poly(3,4-ethylenedioxythiophene) (PEDOT), which provided a wide anodic potential window, high electrical conductivity, and good stability.^37,38^ The bending and contact stability, as well as the electrochemical properties of the reinforced PEDOT/LIG, were characterized, and a synergetic enhancement in both structural stability and electrode kinetics was found. The “artificial enzyme” (*e.g.*, Prussian blue, PB) and natural enzyme (*e.g.*, lactate oxidase, LOx) were immobilized within the porous PEDOT/LIG matrix for the construction of compact and heterostructured flexible skin patch biosensors for the detection of lactate in artificial sweat on a skin model. The advantages of this facile and versatile LIG design approach were further demonstrated by the development of a folded wristband lactate biosensor, as well as a dual-channel electrode system, for simultaneous detection of lactate and glucose.

## Experimental Section

### Materials and Instruments

The details are given in the Supporting Information.

### Fabrication of LIG

Laser irradiation of a polyimide film was performed with a computer-controlled HL40-5g Full Spectrum Laser platform (Full Spectrum Laser LLC, Las Vegas) with a 40 W CO_2_ laser operating with a 1000 ppi resolution in a raster mode under ambient conditions and full laser scan speed (80 in. s^–1^). Overall, 30% of raster power was found to be the threshold. Laser power was then varied from 30 to 100% for the fabrication of LIG (denoted as LIG30–100%) with a fixed laser scan speed (100%). A standalone working electrode (3 mm diameter) and three-electrode system including a working electrode (WE), a reference electrode (RE), and a counter electrode (CE) with a sensing area, a track, and a contact pad were prepared using the optimized power.

### LIG Reinforcement and Immobilization of Artificial and Natural Enzymes

PEDOT was electropolymerized in 0.1 M LiClO_4_ containing a 10 mM EDOT monomer using dynamic potential cycling between 0–1.2 V with a scan rate of 100 mV s^–1^ with different cycles. The optimization of the cycling number for the PEDOT deposition on LIG was evaluated (Figure S1). Then, PB was deposited on the PEDOT/LIG sensing area (PB-PEDOT/LIG) at an optimized constant potential of 0.4 V (Figure S2) in 0.1 M KCl and HCl containing a 5 mM mixture of Fe(CN)_6_^3–^ and FeCl_3_ for 200 s. The immobilization of an enzyme was conducted according to the previously reported literature to achieve high loading of the enzyme with bovine serum albumin (BSA) as an enzyme stabilizer.^39−41^ In brief, an aliquot of a natural enzyme suspension (3 μL) containing 40 mg mL^–1^ LOx and 10 mg mL^–1^ BSA was deposited on PB-PEDOT/LIG and dried at 4 °C for 2 h, followed by the addition of 2 μL of a chitosan solution (1%, in 1% acetic acid). The final enzyme electrode was denoted as LOx/PB-PEDOT/LIG and stored at 4 °C when not in use. To further visualize the loading of the protein/enzyme into the 3D matrix, fluorescein isothiocyanate (FITC)-BSA (1 mg mL^–1^) was chosen as an alternative model protein.

### Integration of the Three-Electrode System as a Skin Patch

The three-electrode system patterned on a flexible polyimide film was assembled into lactate biosensors as wearable devices for detection of lactate in sweat. The electrodes were reinforced with PEDOT as described above. A poly(ethylene terephthalate) (PET) stencil was designed and cut using a cutting device (Brother ScanNcut) to enable Ag/AgCl ink coating within a confined zone using a paintbrush. The ink was then cured on a hotplate at 120 °C for 2 min. The WE was modified with PB, and the enzyme was immobilized on it as described above. Transparent Kapton tape was placed on the track area as a passivation layer and a PC membrane was placed over the three-electrode area as an encapsulation layer.

### Electrochemical Measurements

Electrochemical characterization of LIGs created under different laser powers, electrochemical modification of LIG with PEDOT and PB, and electrochemical measurements of PB-PEDOT/LIG and LOx/PB-PEDOT/LIG standalone working electrodes for H_2_O_2_ sensing and lactate biosensing were conducted using a CompactStat potentiostat (Ivium, Netherlands) with an external platinum (Pt) wire/plate as a CE and Ag/AgCl (3M KCl) as a RE. Electrochemical impedance spectroscopy (EIS) of LIG and PEDOT/LIG standalone working electrodes was performed in 0.1 M KCl containing 5 mM Fe(CN)_6_^3–/4–^ with a frequency range of 100 kHz to 0.1 Hz and a 10 mV amplitude. For the integrated three-electrode system, all of the electrochemical measurements were conducted with the internal Ag/AgCl as RE and LIG as CE. Amperometric lactate biosensing using the integrated three-electrode system was performed using a Sensit Smart potentiostat (PalmSens BV, The Netherlands) connected to a smartphone. Dual-working channel electrode systems were tested using a μStat 400 Bipotentiostat/Galvanostat (DropSens, Spain). Artificial sweat was prepared according to the European standard EN1811:2012 containing sodium chloride (0.5%), potassium chloride (0.1%), and urea (0.1%), and the pH was adjusted to 6.5 with ammonium hydroxide.^42,43^

## Results and Discussion

### Design Concept of a Flexible Skin Patch Biosensor

**Scheme 1:**
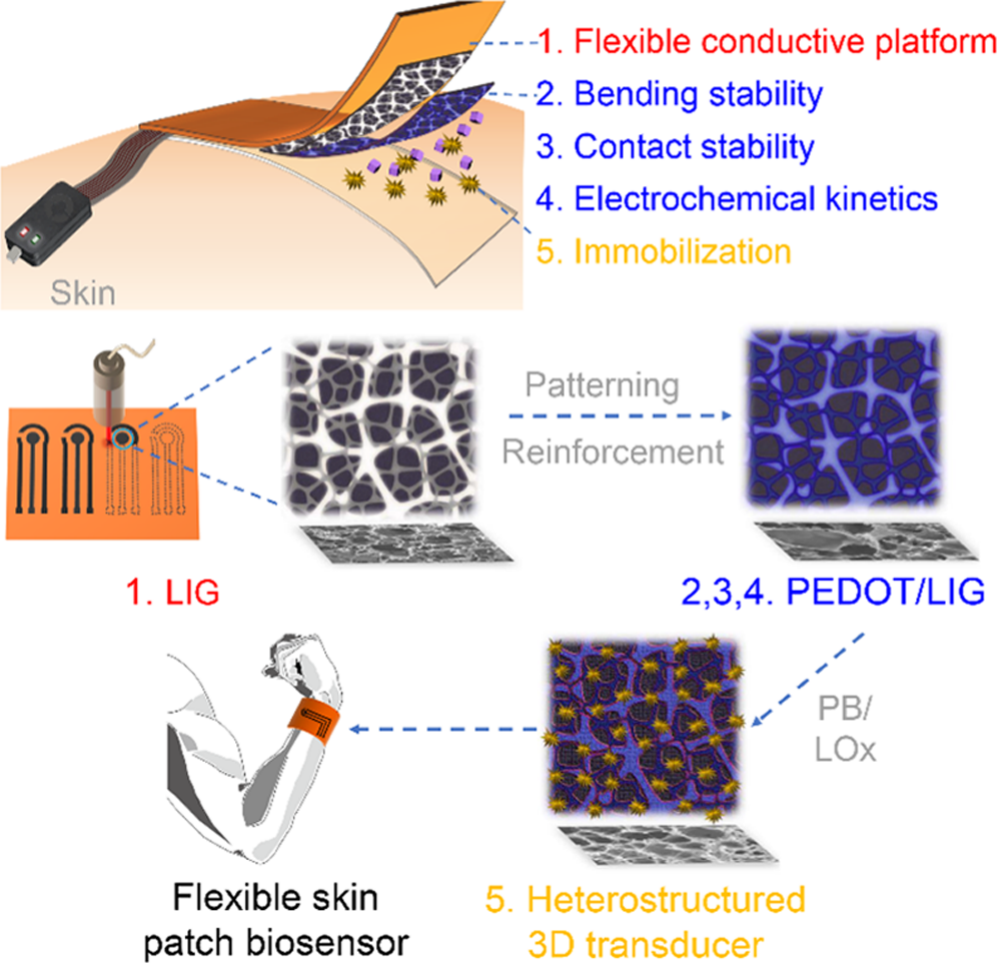
Schematic Diagram of the Flexible Skin Patch Biosensor

Skin patch biosensing devices hold promise for the noninvasive determination of several physiological parameters in perspiration for fitness and health monitoring. Scheme 1 illustrates the design concept of the flexible skin patch biosensor. Localized laser irradiation on a flexible polyimide substrate was employed as a facile and mask-less approach for the design and fabrication of geometrically patterned 3D porous LIG flexible electrodes (1). Then, the LIG served as a macroscopic electrode pattern and a microscopic hard template for the nanodeposition of PEDOT, which reinforced the structural stability of the hierarchically interconnected graphene network as a conductive polymeric binder, resulting in improved bending stability, contact stability, and enhanced electrochemical kinetics of the overall PEDOT/LIG (2, 3, 4). The interconnected pores of PEDOT/LIG functioned as a 3D porous host matrix for the high loading immobilization of artificial and natural enzymes, resulting in a compact and heterostructured 3D transducer as an innovative skin patch biosensor (5).

### Optimization and Characterization of LIG

**Figure 1:**
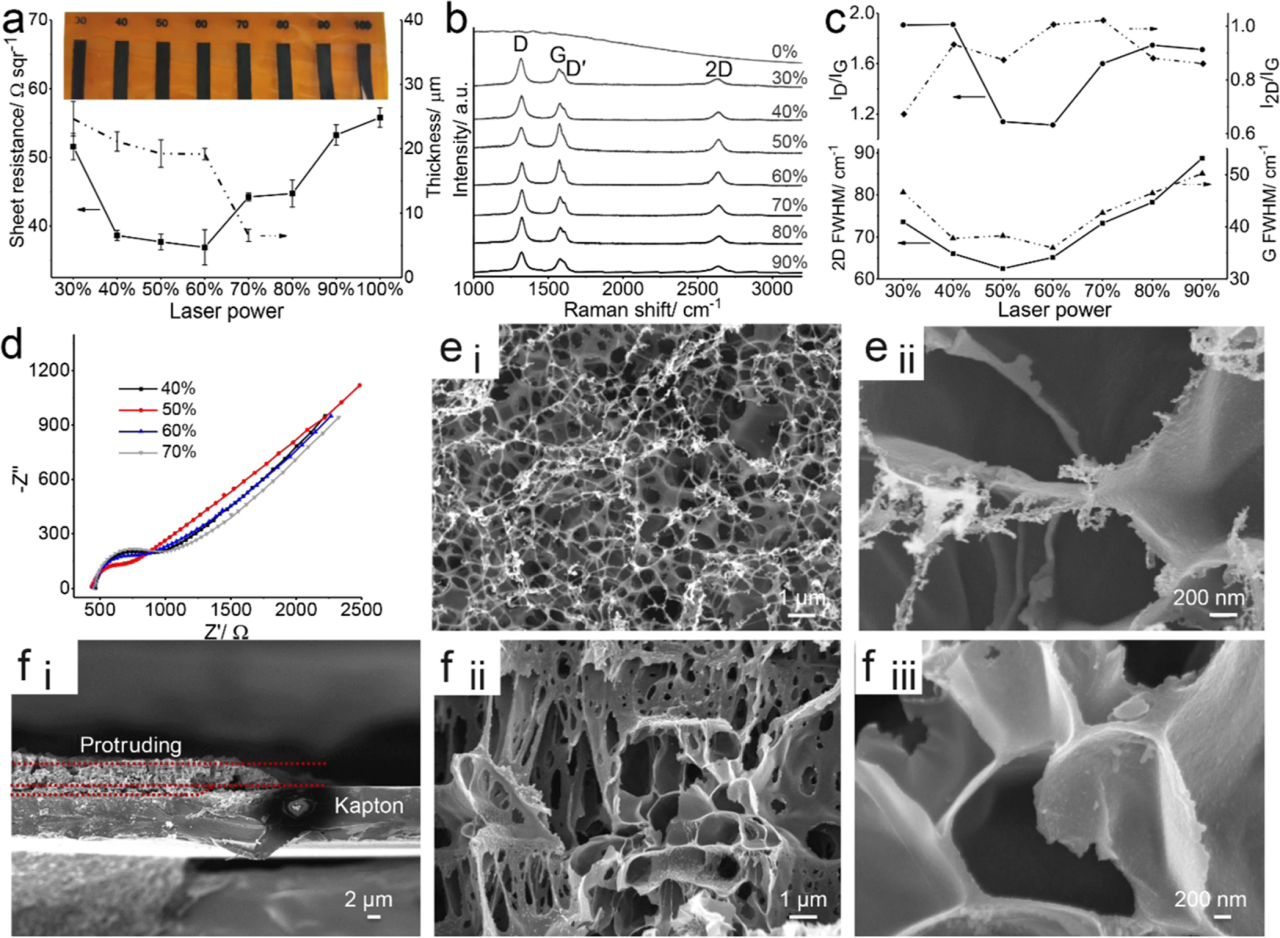
(a) Sheet resistance and thickness of LIG using different laser powers over the range 30–100%; the inset is the digital image of LIG30–100% strips. (b) Raman spectra of LIG with different laser powers over the range 30–90% and the original polyimide film. (c) Summary of the intensity ratio (*I*_D_/*I*_G_, *I*_2D_/*I*_G_) and FWHM of 2D and G bands. (d) EIS of LIG40–70% in 5 mM Fe(CN)_6_^3–/4–^ in 0.1 M KCl. The top-view (e) and cross-sectional (f) SEM images of LIG50% at different magnifications.

The precise laser power is critical for the conductivity and thickness of LIG since low laser power cannot reach the threshold to realize the carbonization/graphitization, while extreme high power would cause the ablation of the entire material. As shown in the digital image of LIG30–100% in Figure 1a (inset), 30% laser power was found to be the threshold power for initial carbonization/graphitization of a polyimide film. Detailed optical images (Figure S3a) show the incompletely covered graphene film with holes on the polyimide at low power (LIG30%). The graphene film appeared darker in color and as a more compact film with power increasing from 30 to 50%, while a further increase in laser power from 60 to 100% resulted in the development of vertical cracks and final fracture of the film with 100% laser power. The thickness of the LIG protruding at different laser powers (Figure 1a) displayed a slight decrease from 24.6 ± 2.7 μm for LIG30% to 19.2 ± 1.5 μm for LIG60%, whereas it sharply dropped to 6.6 ± 0.9 μm for LIG70%, and the thickness of LIG80–100% was undetectable. The decrease of the thickness with an increase in laser power could be attributed to (1) higher laser power causing the dissociation and ablation of deep-seated polyimide molecules and (2) faster evolution and release of the gaseous products inside the pores, resulting in the destruction of the graphene structure and splashing of graphene snippets, which is confirmed by scanning electron microscopy (SEM) with the appearance of graphene snippets on the polyimide film without laser exposure adjacent to the graphene track (Figure S3b). In addition, the sheet resistance of the LIG demonstrated a decreasing trend from 51.6 ± 2.0 Ω sqr^–1^ (LIG30%) to 36.9 ± 2.6 Ω sqr^–1^ (LIG60%) with the increase of laser power over the range of 30–60%, while a further increase of laser power led to an apparent increase in sheet resistance to 55.8 ± 1.4 Ω sqr^–1^ (LIG100%). The calculated electrical conductivity of the resulting LIG under increasing increments of laser power (Figure S4) exhibited an increasing trend with the maximum value of 34.4 S cm^–1^ for LIG70%, which is in the same magnitude as previously reported LIG.^10,44,45^

The LIG under different laser powers was further characterized by Raman spectroscopy (Figure 1b). No obvious bands were noted for polyimide. Once exposed to a laser, three main bands appeared for all of the LIG30–90%, including a D band at 1324 cm^–1^ related to the disordered structure of graphene, a G band at 1577 cm^–1^ due to the *E*_2g_ mode at the Γ-point arising from stretching of a sp^2^ C–C bond in graphitic materials with a shoulder D′ band (1603 cm^–1^) for randomly distributed impurities or surface charges, and a 2D band at 2636 cm^–1^ for the stacking order of graphene layers.^46,47^ The intensity ratios of the D and 2D bands compared to the G band are usually used as a sensitive metric for the degree of disorder and stacked graphene layers, respectively.^47^ The statistical analysis of *I*_D_/*I*_G_ and *I*_2D_/*I*_G_ ratios as well as the full-width at half maximum (FWHM) of 2D and G bands as a function of laser power, are summarized in Figure 1c. A lower value of *I*_D_/*I*_G_, a higher value of *I*_2D_/*I*_G_, and narrower 2D and G FWHM for LIG50–60% than those of low (30%, 40%) and high (70–100%) laser powers indicate the low degree of disorder and high crystalline size with a few-layered graphene structure.^9^

The electrochemical properties of LIG40–70% were investigated by cyclic voltammetry and electrochemical impedance spectroscopy (EIS). All cyclic voltammograms (CVs) for LIG40–70% electrodes showed typical quasi-reversible peaks with a Fe(CN)_6_^3–/4–^ probe (Figure S5a,b), while LIG50% possessed the lowest peak-to-peak difference (128 mV) and the highest peak height current (95.7 μA). The Nyquist plots from EIS in Figure 1d exhibited a semicircle feature in the high-frequency region related to the charge-transfer resistance (*R*_ct_) and a straight line feature in the low-frequency region corresponding to the semi-infinite diffusion-controlled process. The equivalent circuit and detailed fitting parameters are summarized in Figure S5c. Of these, the lowest Rct value of 325 Ω for LIG50% suggests a fast electron-transfer rate, which is consistent with the results from cyclic voltammetry. Therefore, 50% laser power was chosen as the optimized parameter for LIG electrode fabrication hereinafter.

The surface morphologies of LIG50% and the pristine polyimide film were examined using SEM. The top-view SEM image of the pristine polyimide film (Figure S3c) displays a flat and smooth surface. The irradiation of laser converted the smooth polyimide into an ordered porous honeycomb-like structure, as shown in Figure 1e(i). The high-magnification SEM image for LIG50% shown in Figure 1e(ii) shows the open macropores formed by the interconnected graphene layers. In addition, there are residues of graphene snippets on the edge of graphene walls from high-power-induced photothermal and ablation processes, which is also seen on a polyimide film without laser exposure adjacent to the graphene track (Figure S3b). The cross-sectional SEM image shown in Figure 1f(i) confirms the conversion of polyimide into graphene protruding with a height of ∼20.5 μm above the base polyimide level, which is consistent with the result from the surface profiler. The graphene structure penetrates ∼8 μm into polyimide due to the ablation effect. High magnification of the cross-sectional SEM images shown in Figure 1f(ii, iii) reveals a similar porous graphene structure as the top view and large vertical channels alongside the path of the laser, which might be ascribed to rapidly liberated gaseous products throughout the film in the vertical direction alongside the laser. All of the SEM results indicate the successful formation of a 3D porous graphene architecture on the polyimide.

### PEDOT Reinforcement of LIG

**Figure 2:**
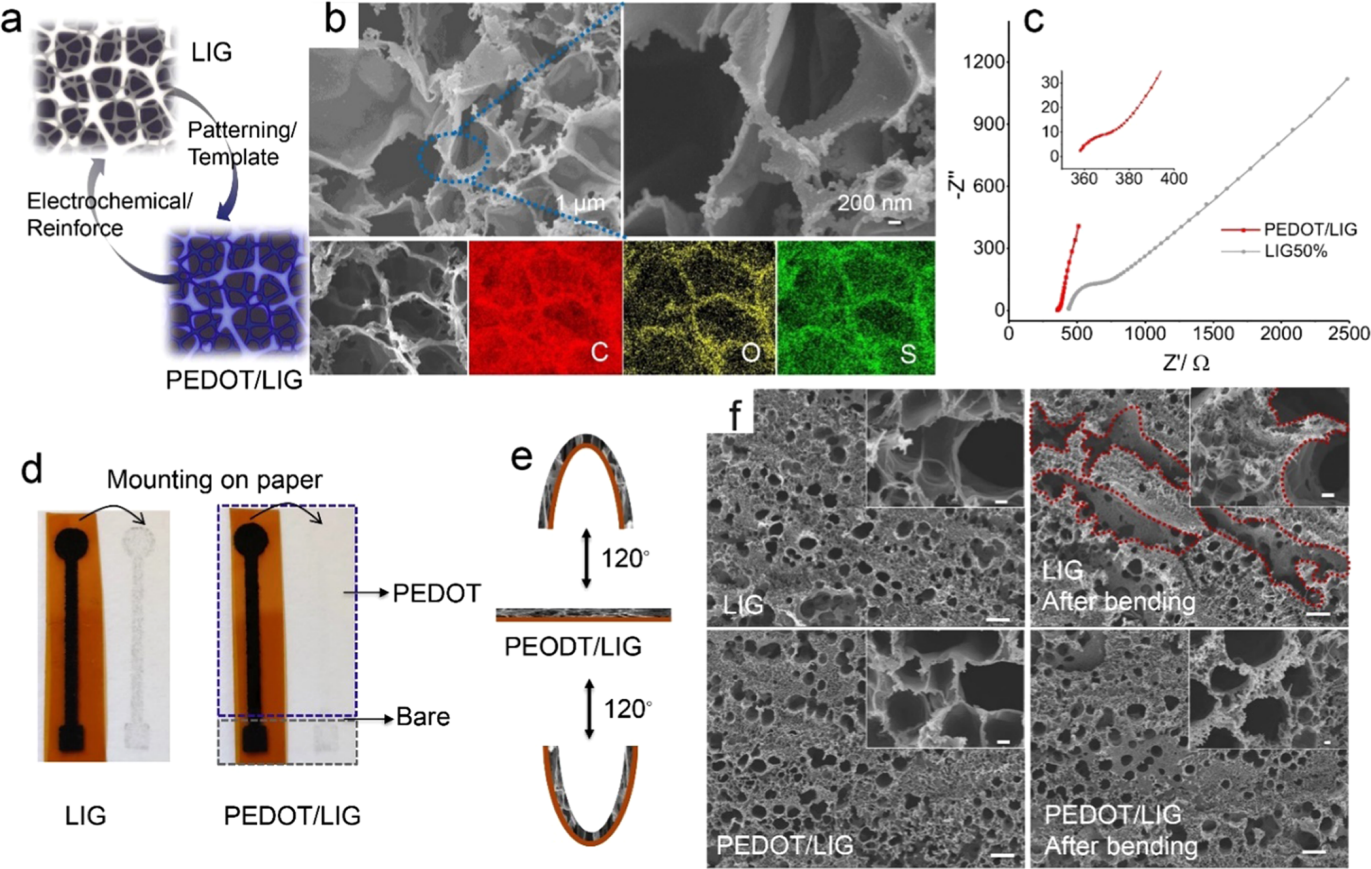
(a) Schematic illustration of patterning PEDOT on LIG; (b) SEM images of PEDOT/LIG and EDS mapping; (c) EIS of PEDOT/LIG and bare LIG50% in 5 mM Fe(CN)_6_^3–/4–^ in 0.1 M KCl; (d) digital image of mounting LIG and PEDOT/LIG onto paper; (e) schematic diagram of the structural stability test; (f) SEM images of LIG (up) and PEDOT/LIG (down) before (left) and after (right) bending; the scale bar is 20 μm and the inset scale bar is 1 μm.

The localized laser irradiation method provides a facile and mask-less approach for the design and fabrication of various geometrically patterned LIG flexible electrodes, including a standalone working electrode, a three-electrode system, as well as a dual-channel electrode and a multichannel electrode system, as shown in Figure S6. However, LIG with its porous structure is fragile in nature and incompatible with the flexible polyimide substrate, which can result in failing and cracking of the LIG film. To overcome this problem, PEDOT was employed as a conductive polymeric binder to reinforce the favorable 3D porous morphology of the LIG structure via adhesion and binding effects (Figure 2a). Figure 2b shows the nanodeposition of PEDOT alongside the well-maintained porous structure, and the energy-dispersive X-ray spectroscopy (EDS) mapping of C, O, S suggests the good distribution of PEDOT on the porous LIG. The mask-free patterned LIG on a flexible polyimide film served as not only a macroscopic pattern for the deposition of PEDOT into the designed electrode shape but also as a microscopic hard template for the nanodeposition of PEDOT.

After deposition of PEDOT, the *R*_ct_ value from the Nyquist plot (Figure 2c) exhibited an approximately 15-fold decrease from 325 Ω (bare LIG) to 21 Ω (PEDOT/LIG), which reveals the improved electrode kinetics originating from the excellent electronic/ionic conductivity of PEDOT (Figure S5c). In addition, the PEDOT functions as an ideal polymer binder to reinforce the porous graphene structure with effectively minimized failing of LIG while mounting onto paper (Figure 2d). On the contrary, the bare LIG left black-colored LIG residues after mounting onto a paper strip. To further evaluate the reinforcement effect of PEDOT during deformation, the LIG and PEDOT/LIG were bent by 120° in two directions 200 times (Figure 2e). SEM images (Figure 2f) of bare LIG show the generation of cracks in the interconnected graphene structure after bending. As a comparison, no obvious cracks were noted in PEDOT/LIG before and after bending, indicating the improved structural strength and stability due to the reinforcement effect of the polymeric binder PEDOT. The reinforced structural stability of the interconnected graphene network containing PEDOT was also confirmed by the CV characterization in 0.1 M KCl and 5 mM Fe(CN)_6_^3–/4–^ (Figure S7) with well-retained CV curves, indicating that the electrochemical properties of the PEDOT/LIG are less affected by bending stresses.

### Artificial and Natural Enzyme-Coupled PEDOT/LIG Heterostructured Transducer

**Figure 3:**
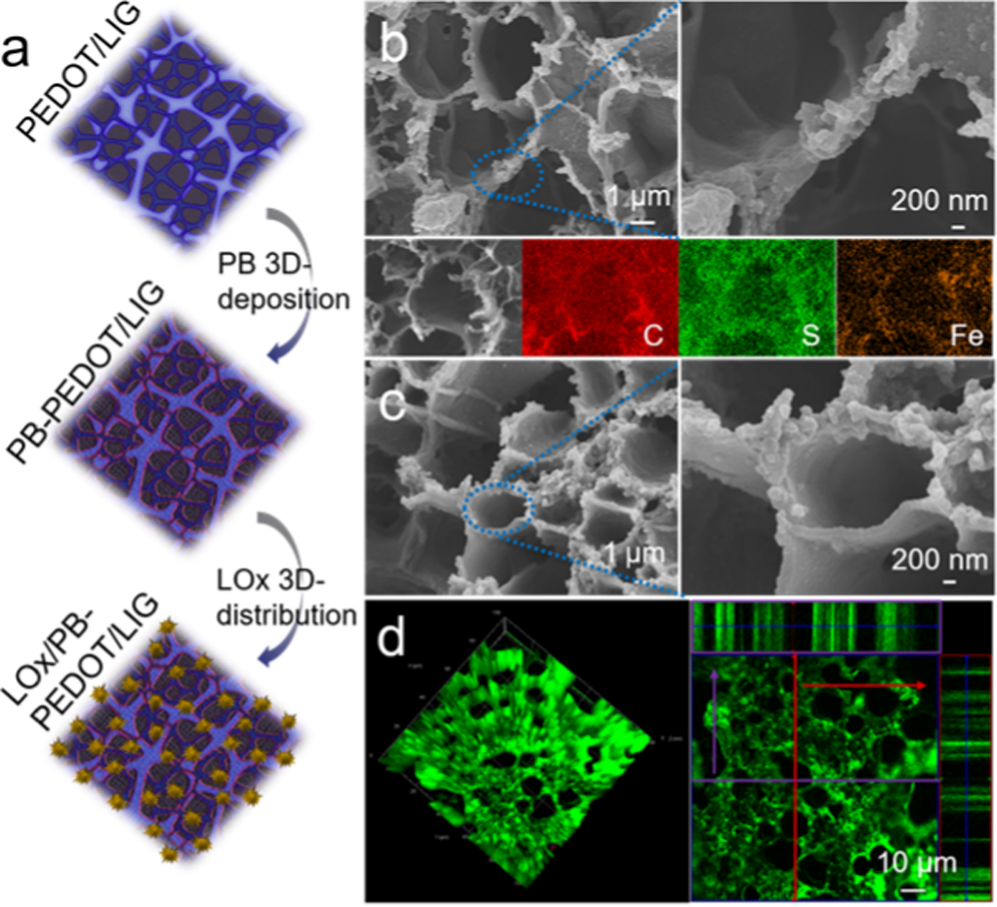
(a) Schematic illustration of PB deposition and LOx loading on porous PEDOT/LIG; (b) SEM images of PB-PEDOT/LIG, and its EDS mapping of key elements C, S, and Fe; (c) SEM images of LOx/PB-PEDOT/LIG; and (d) 3D (left) and orthogonal-section (right) confocal images of FITC-BSA-immobilized PB-PEDOT/LIG.

Porous PEDOT/LIG provides an excellent porous substrate for the fabrication of a compact and heterostructured 3D transducer, as shown in Figure 3a. To establish efficient signal transduction in biosensing, PB was deposited on the PEDOT/LIG as a simple and robust catalyst for the reduction of the enzymatic intermediate H_2_O_2_. As shown in Figure 3b, the deposition of PB did not affect the porous structure of the LIG electrode. The appearance of the signature Fe element in EDS mapping verified the successful deposition of PB with the homogeneous distribution. Detailed information of other key elements (*e.g.*, K, N, Cl) are shown in Figure S8. Moreover, the stable and effective immobilization of enzymes is another important prerequisite for an effective biosensing device with good sensitivity. SEM images in Figures 3c and S9 show the distribution of LOx and its stabilizer BSA immobilized within the interconnected pores of the PB-PEDOT/LIG, forming a compact and heterostructured transducer. The EDS spectrum (Figure S9) further illustrates the effective loading of LOx/BSA molecules with the nitrogen increasing from 2.5 wt % for PB-PEODT/LIG to 12.4 wt % for LOx/PB-PEDOT/LIG. FITC-BSA was used to visualize the loading of biomolecules into the PB-PEODT/LIG matrix. The 3D projection of the confocal image shown in Figure 3d (left) shows strong and spatially specific fluorescence emission owing to the high loading of protein molecules into the 3D porous PB-PEDOT/LIG structure. The 2D orthogonal-section confocal image (Figure 3d right) further indicates the loading of FITC-BSA alongside the wall of the 3D graphene pores. These results verified the successful application of PEDOT/LIG as a host matrix for the loading of both artificial and natural enzymes to construct the heterostructured 3D transducer.

**Figure 4:**
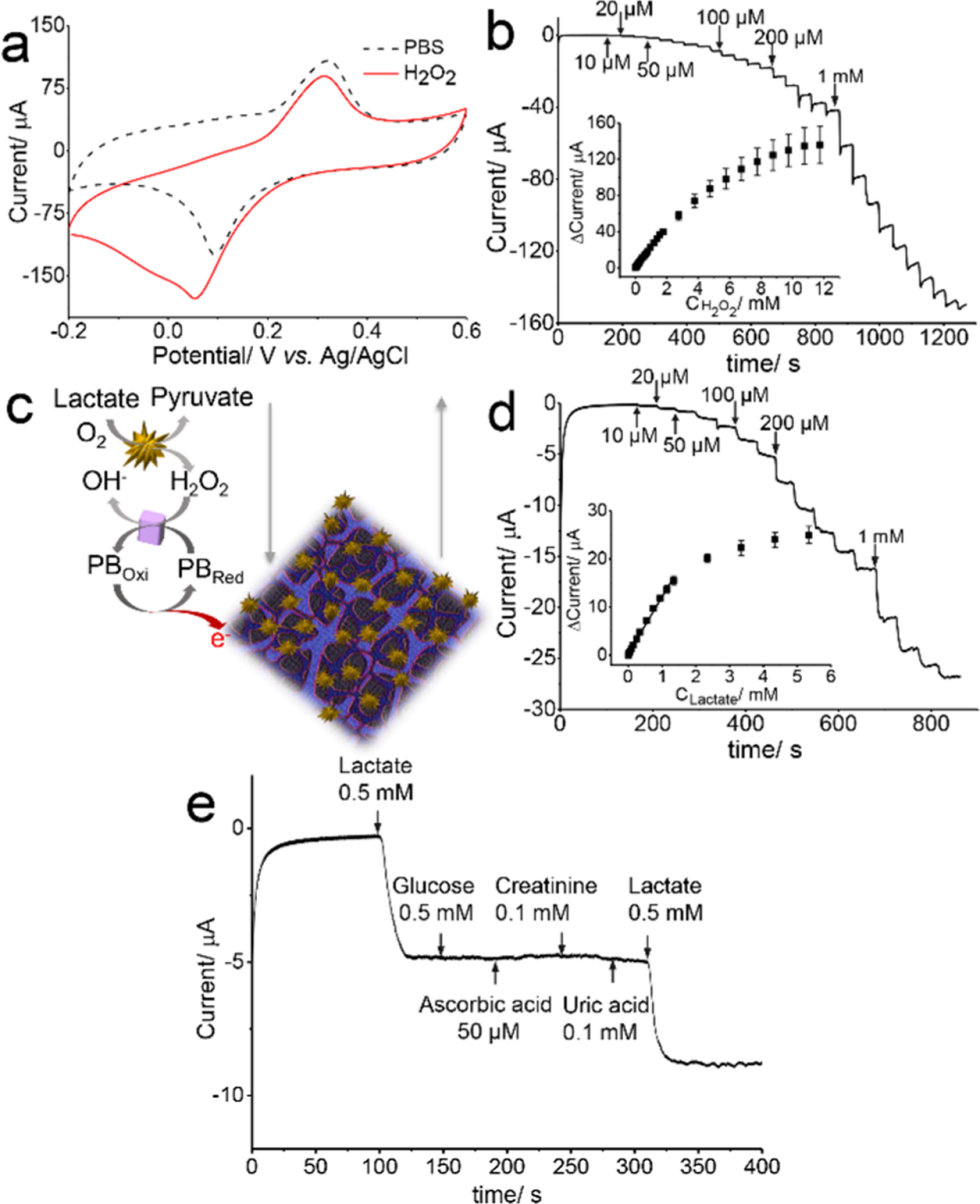
(a) CVs of PB-PEDOT/LIG to 5 mM H_2_O_2_ in 0.1 M PBS (pH = 6.4), the scan rate of 50 mV s^–1^; (b) current–time curve of PB-PEDOT/LIG to successive addition of H_2_O_2_ in 0.1 M PBS (pH = 6.4) at 0 V, the inset is the calibration curve, *n* = 3; (c) schematic of lactate biosensing and signal transduction at LOx/PB-PEDOT/LIG; (d) current–time response curve of LOx/PB-PEDOT/LIG to successive addition of lactate in 0.1 M PBS (pH = 6.4) at 0 V, the inset is the calibration curve, *n* = 3; and (e) interference study of the LOx/PB-PEDOT/LIG electrode.

The electrochemistry of H_2_O_2_ and lactate at the heterostructured 3D transducer was evaluated. Figure 4a shows the CVs of PB-PEDOT/LIG in the absence and the presence of 5 mM H_2_O_2_ in 0.1 M PBS (pH = 6.4). In the absence of H_2_O_2_, a pair of quasi-reversible peaks was found due to the redox couple “Fe^III^ ⇆ Fe^II^” in PB with a reduction peak height current of 83.03 μA, while after the addition of H_2_O_2_, the reduction peak height current increased dramatically to 147.2 μA due to the electrocatalytic effect of PB toward H_2_O_2_ reduction. The current–time response of H_2_O_2_ at the PB-PEDOT/LIG electrode was measured at 0 V with successive addition of H_2_O_2_. As can be seen from Figure 4b, PB-PEDOT/LIG responded to each addition of H_2_O_2_, reaching a steady-state current within 10 s. The corresponding calibration curve (Figure 4b inset) was linear over a H_2_O_2_ range of 0.01–1.76 mM (*R*^2^ = 0.999) with a high sensitivity of 24.33 μA mM^–1^ and a low limit of detection (LOD) calculated to be 2.5 μM (3σ/sensitivity).

In the light of the good analytical performance of the PB-PEDOT/LIG electrode for sensing H_2_O_2_, LOx was immobilized on the electrode to create an electrochemical biosensor for lactate (Figure 4c). Figure 4d shows a typical current–time curve of the LOx/PB-PEDOT/LIG biosensor response to successive addition of lactate at 0 V. A stepwise increase in the current response was observed for each successive addition of lactate, reaching 95% of the steady-state current within 10 s. The current response (Figure 4d inset) was linear with the lactate concentration over the range of 0.01–1.35 mM (*R*^2^ = 0.995) with a sensitivity of 11.83 μA mM^–1^. The LOD was calculated to be 6.8 μM (3σ/sensitivity). The analytical performance of the electrochemical sensing of H_2_O_2_ at PB-PEDOT/LIG and biosensing of lactate at LOx/PB-PEDOT/LIG were comparable with, or in some cases better, than other PB-based materials and LOx-based electrodes described in the literature (Table S1) with respect to sensitivity and the linear range. The good analytical performance is likely facilitated by the (1) porous LIG electrode with a high surface area, (2) synergetic morphological and electrochemical reinforcement of LIG using PEDOT, and (3) a heterostructured transducer with high loading of artificial and natural enzyme molecules into the porous PEDOT/LIG. Moreover, the lactate biosensor showed no detectable response to common interfering electroactive species in sweat including glucose, ascorbic acid, creatinine, and uric acid (Figure 4e), indicating its good selectivity.

### Flexible Skin Patch Lactate Biosensor

**Figure 5:**
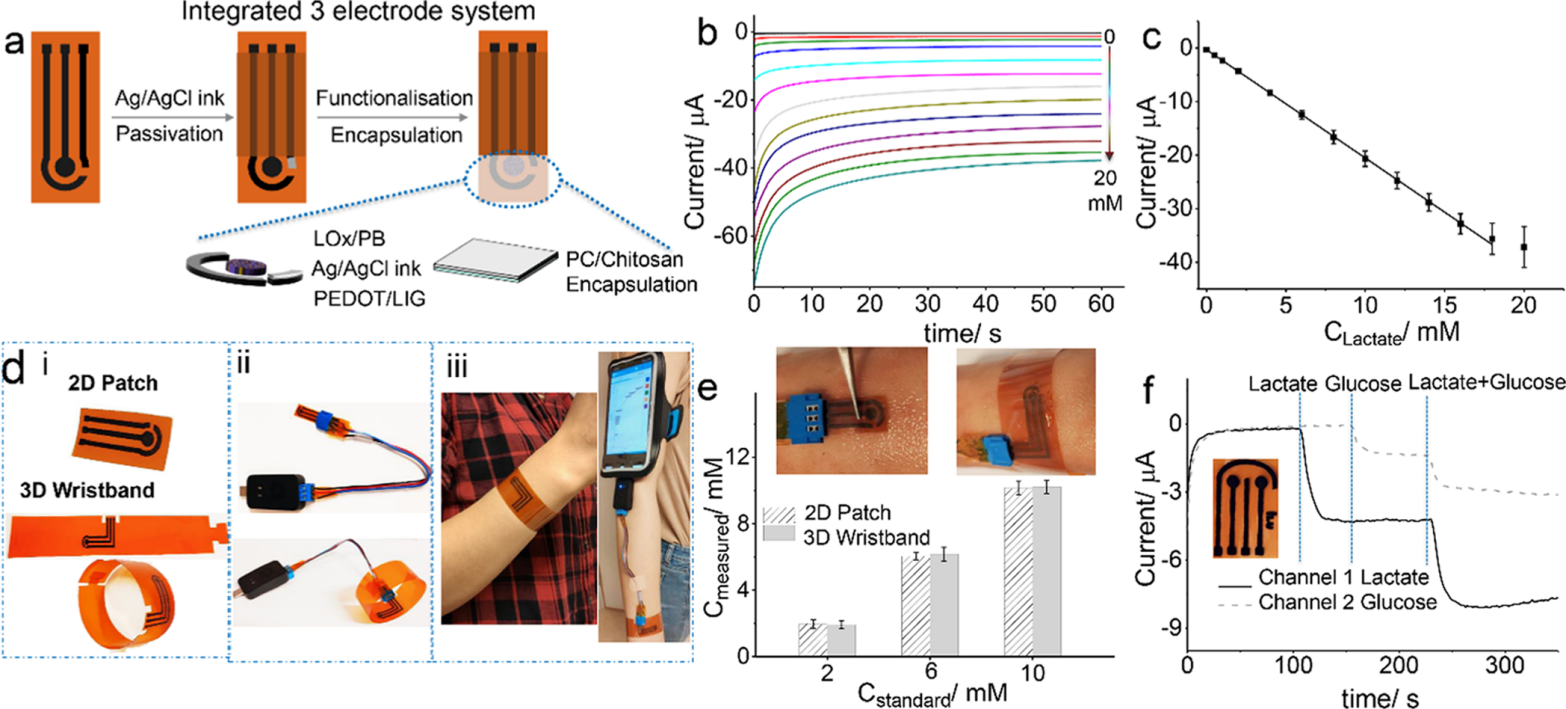
(a) Integration of the three-electrode system; (b) amperometric response of the integrated three-electrode system to increasing concentration of lactate in the range of 0–20 mM in artificial sweat; (c) corresponding calibration curve, *n* = 3; (d) digital image of the 2D patch and the 3D wristband (i), connection with a cable and miniaturized potentiostat (Sensit Smart) (ii), and 3D wristband conformed for wearing (iii); (e) comparison of the 2D patch and the 3D wristband for lactate detection in artificial sweat on the skin model; and (f) integration of the dual-channel electrode system for simultaneous detection of lactate and glucose.

An integrated flexible skin patch lactate biosensor was further developed based on the heterostructured LOx/PB-PEDOT/LIG transducer, which consisted of a custom-built integrated three-electrode system, as shown in Figure 5a. We examined the stability of the integrated three-electrode system using a Fe(CN)_6_^3–/4–^ probe and observed no obvious decay or deviation of the redox peaks after 1000 cycles, indicating the good stability of the custom-built Ag/AgCl reference electrode (Figure S10). Characteristic Nyquist plots (Figure S11) of the integrated three-electrode system based on PEDOT/LIG with a small Rct value (11 Ω) supports the feasibility of the custom-built integrated three-electrode system for the fabrication of a flexible skin patch lactate biosensor. Moreover, the analytical dynamic window of the LOx/PB-PEDOT/LIG working electrode was adjusted by the addition of a diffusion layer consisting of PC/chitosan for the detection of physiological lactate concentrations.^48^

The integrated skin patch lactate biosensor was operated at an optimized potential of −0.1 V for amperometric lactate detection to deliver a high current response to lactate and low background current, following evaluation of the current response at different potentials from −0.25 to 0.1 V (Figure S12). The dynamic concentration range of the integrated skin patch biosensor in response to lactate in artificial sweat was then investigated. The amperometric response (Figure 5b) increased gradually with increasing concentration of lactate over the range of 0–20 mM in artificial sweat. As shown in the calibration curve in Figure 5c, the integrated skin patch biosensor exhibited a well-defined linear portion of the current response to lactate over the range of 0–18 mM (*R*^2^ = 0.998) with a high sensitivity of 2.23 μA mM^–1^. The analytical performance of the integrated skin patch lactate biosensor based on PEDOT/LIG was compared with other reported amperometric lactate biosensors (summarized in Table S2), illustrating its good performance with respect to sensitivity.

The practical applications of the flexible skin patch-based lactate biosensor were further validated by the detection of lactate in sweat on a skin model with two different designs, *i.e.*, a 2D patch and a 3D wristband, as shown in Figure 5d (i–iii), in which the 2D patch can be attached to the skin surface and the long strip can be scrolled to a 3D wristband for intimate contact to the skin. Moreover, Figure 5d (iv) shows a 3D wristband worn on a volunteer’s wrist connected to a portable miniaturized potentiostat (Sensit Smart) and cell phone. The performance of these two different *in vitro* wearable designs was evaluated by their ability to detect lactate concentrations of 2, 6, and 10 mM sprayed on the skin model. It can be seen from Figure 5e (amperometric curves in Figure S13) that the measured concentration of lactate was highly consistent with the known concentration, and no obvious difference was found between the 2D patch and the 3D wristband. It should be noted that *ex vivo* experiments have not yet been carried out.

To expand the potential applications of this flexible and wearable biosensing platform, glucose oxidase (GOx) was immobilized on an electrode to enable glucose detection, and the current response (Figure S14) was found to be linear to the glucose concentration over the range of 0.01–2.56 mM (*R*^2^ = 0.995) with a sensitivity of 3.87 μA mM^–1^. A dual-channel electrode system was then integrated for simultaneous monitoring of glucose and lactate. As shown in Figure 5f, channel 1 (lactate) and channel 2 (glucose) responded to 0.5 mM lactate and 0.5 mM glucose, respectively, without any apparent cross-talk. The addition of a mixture of lactate/glucose resulted in the same relevant current response in each channel to that obtained with the individual additions. Such an integrated flexible and wearable biosensing platform can be potentially expanded for multichannel and noninvasive monitoring of several physiological analytes in sweat, such as glucose, lactate, and cortisol or even pH and electrolytes. In addition, future integration of the flexible sensing platform with soft epidermal microfluidics is promising for sweat collection and its delivery to the sensing area for more accurate and reliable detection.^49^

## Conclusions

In summary, we have demonstrated conducting polymer-reinforced graphene as a heterostructured 3D transducer in a flexible skin patch biosensor. This platform exhibited good bending properties and contact stability coupled with improved electrochemical kinetics and biochemical immobilization. The mask-free laser irradiation method to produce graphene on polyimide under the optimized conditions resulted in a flexible LIG conductive platform, which then served as an electrode pattern and porous template for the deposition of the conducting polymer PEDOT. PEDOT improved the structural stability as a conductive polymeric binder by reinforcing the porous structure as well as the electrochemical properties of the LIG material due to its intrinsic electronic/ionic conductivity. PEDOT/LIG provided a highly flexible platform for the fabrication of compact and heterostructured 3D transducers for skin patch (or wristband) biosensors. Amperometric lactate biosensing over a wide linear range and with high sensitivity was demonstrated in artificial sweat. Simultaneous monitoring of glucose and lactate using a dual-channel system showed no apparent cross-talk. Such an integrated flexible and wearable biosensing platform can be potentially expanded for multichannel and noninvasive monitoring of several physiological parameters in perspiration for fitness and health monitoring.

## Supporting Information Available

Details of materials and instruments for characterizations; optimization of PEDOT and PB depostion; optical and SEM images of LIG strips and polyimide; calculated conductivity of LIG; cyclic voltammograms of LIG; EIS fitting models; digital images of patternable electrode systems; bending stability of LIG and PEDOT/LIG; EDS of PB-PEDOT/LIG; SEM and EDS of LOx/PB-PEDOT/LIG; stability test of Ag/AgCl ink paste as an internal reference electrode; Nyquist plot of integrated three-electrode system based on PEDOT/LIG; ; optimization of potential for lactate biosensing; amperometric curve of the 2D patch/3D wristband; glucose biosensing; comparison of biosensing performance with the literature. (PDF)
