# Supplementary material for: Conducting Polymer-Reinforced Laser-Irradiated Graphene as a Heterostructured 3D Transducer for Flexible Skin Patch Biosensors
Source: ACS Appl Mater Interfaces. 2021 Nov 2;13(45):54456–65. doi: 10.1021/acsami.1c13164 (PMC8603349; doi:10.1021/acsami.1c13164)
Supplement: Supplementary file 1 — am1c13164_si_001.pdf [file am1c13164_si_001.pdf]

Supporting information

Conducting Polymer Reinforced Laser-irradiated Graphene as a Heterostructured 3D Transducer for Flexible Skin Patch Biosensors

Lingyin Meng, Anthony P. F. Turner[+]*, and Wing Cheung Mak**

Biosensors and Bioelectronics Centre, Division of Sensor and Actuator Systems, Department of Physics, Chemistry and Biology, Linköping University, SE-581 83 Linköping, Sweden

[+] Present address: Professor Emeritus, SATM, Cranfield University, Bedfordshire, MK430AL, UK.

*Corresponding author.

E-mail address: wing.cheung.mak@liu.se (W.C. Mak).

Materials

Polyimide film (thickness 75 µm) and Kapton masking tape were purchased from RS Components AB (Sweden). Potassium ferricyanide ($K_3[Fe(CN)_6]$), potassium ferrocyanide ($K_4[Fe(CN)_6]$), 3, 4-ethylenedioxythiophene (EDOT), lithium perchlorate ($LiClO_4$), Iron(III) chloride ($FeCl_3$), hydrogen peroxide (H_2O_2), sodium L-lactate, albumin-fluorescein isothiocyanate conjugate (FITC-BSA), bovine serum albumin (BSA), chitosan and glucose oxidase (GOx, 168 U mg^{-1}) were purchased from Sigma-Aldrich (USA). Lactate oxidase (LOx, 106 U mg^{-1}) was bought from Toyobo (Japan). Polycarbonate membrane (PC, 0.2 µm) was obtained from Avanti Polar Lipids, Inc. (USA). Silver/silver chloride (Ag/AgCl) was purchased from Dupont (USA). All chemicals were of analytical grade and used without any further treatment. Deionised water from a MilIi-Q System was used throughout.

Instruments

Thickness measurements for the LIG strips at different laser power were performed on a Dektak 6 M surface profilometer (Veeco Instruments Inc., USA) with a scanning distance of 7000 µm. Sheet resistance was measured with a 4-point probe sheet resistivity meter (Model 280C, Four Dimensions Inc., USA). Optical images of LIG were collected with a SMZ1000 stereomicroscope (Nikon Metrology Inc., Japan). Scanning electron microscopy (SEM) images were recorded using a LEO 155 Gemini (Zeiss, Germany) electron microscope. Fourier transform infrared (FTIR) spectroscopy was performed using a VERTEX spectrometer (Bruker, USA) equipped with an attenuated total reflection (ATR) measuring cell. Raman spectra were acquired with a LabRAM HR 800 Raman spectrometer (Horiba Jobin Yvon, France) using a 660 nm 5 mW laser. Chemical composition and mapping were determined employing energy-dispersive X-ray spectroscopy (EDS, Oxford Instruments). The distribution of FITC-BSA in the 3D matrix was studied using a LSM 700 confocal microscope (Zeiss, Germany).

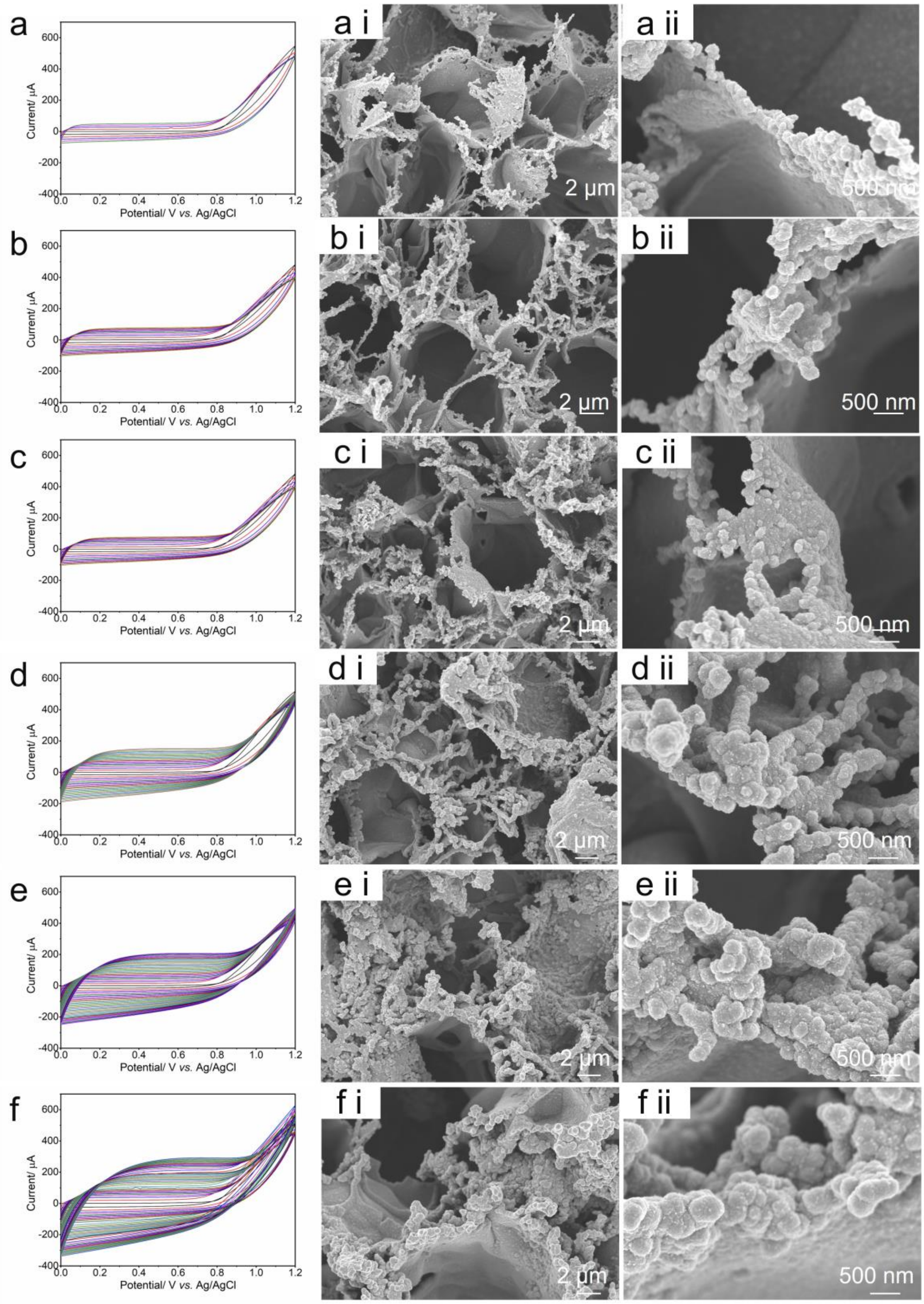

Figure S1. Optimization of PEDOT electrochemical deposition on LIG with different cycling number and the corresponding SEM images in different magnifications. (a) 5 cycles, (b) 10 cycles, (c) 15 cycles, (d) 20 cycles, (e) 30 cycles and (f) 40 cycles.

The CVs in Figure S1 a-f demonstrate the potentiodynamic polymerization of PEDOT on LIG with different cycles. 1.2 V was used for the polymerization of PEDOT with high efficiency while avoiding overoxidation, which caused notable changes in electrochemical and morphological properties of the PEDOT film (*e.g.*, cracks, delamination and decreased conductivity)[1-3]. In all CV curves, we observed the onset potential around 0.8 V in the forward scan and a current crossover at about 0.85 V in the reverse scan for the 1st cycle, which represents the characteristic nucleation process of PEDOT[4-5]. Upon the successive cycles, the current showed a notable increase, implying a successful gradual growth process of PEDOT on the electrode surface. As the number of cycles increased, the charging current (CV box) for the final cycle curve increased due to the accumulation of PEDOT on LIG.

The electropolymerization of PEDOT on LIG with different cycles was examined by SEM. As can be seen in Figure S1, the porous structure of the LIG was well-maintained for 5 and 10 cycles. Further increase of the cycling number resulted in the accumulation of PEDOT on LIG, which to some extent blocked the 3D porous structure. Taking into consideration of the PEDOT reinforcement effect with a specific amount and the well-maintained 3D porous structure for effective immobilization of enzyme, 10 cycles was chosen for the electropolymerization of PEDOT.

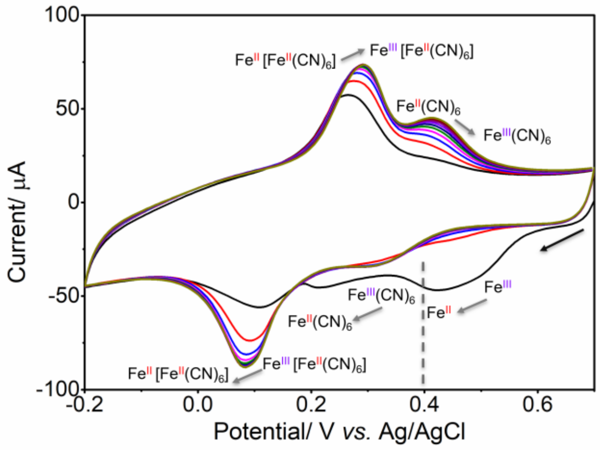

Figure S2. CVs of PEODT/LIG electrode in 0.1 M KCl and HCl containing a 5 mM mixture of $K_3[Fe(CN)_6]$ and $FeCl_3$.

A constant potential for PB deposition was selected/optimized from the CVs of PEDOT/LIG in 0.1 M KCl and HCl containing a 5 mM mixture of $Fe^{III}(CN)_6^{3-}$ and $Fe^{III}Cl_3$. On the first scan (reverse direction from 0.7 to -0.2 V), the Fe^{III} was reduced to Fe^{II} at around 0.4 V, which further reacted with $Fe^{III}(CN)_6^{3-}$ for bulk precipitation of Prussian blue. Further cycling also resulted in the reduction of $Fe^{III}(CN)_6^{3-}$ to $Fe^{II}(CN)_6^{3-}$ at around 0.22 V and the reduction peak for Prussian blue, $Fe^{III}Fe^{II}$ $(CN)_6^{3-}$), into Prussian white, $Fe^{II}Fe^{II}$ $(CN)_6^{3-}$) at around 0.19 V. 0.4 V was chosen for PB deposition under constant potential because of the: 1) efficient reduction of Fe^{III} species; 2) bulk precipitation of Prussian Blue; and 3) less regular polycrystal structure of Prussian blue under lower potential (0.2 V) because both $Fe^{III}(CN)_6^{3-}$ and Fe^{III} species are reduced.[6]

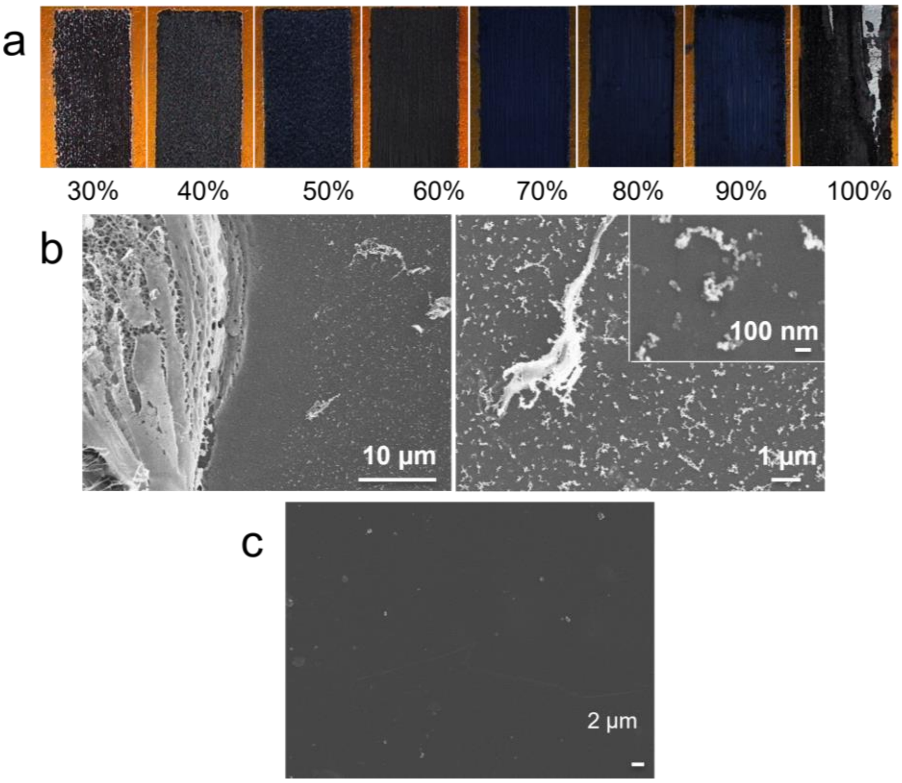

Figure S3. (a) Optical images of LIG strips using different laser power over the range 30–100%; (b) SEM image of LIG50% showing the appearance of graphene snippets on polyimide film without laser exposure adjacent to the graphene track; (c) SEM image of pristine polyimide film.

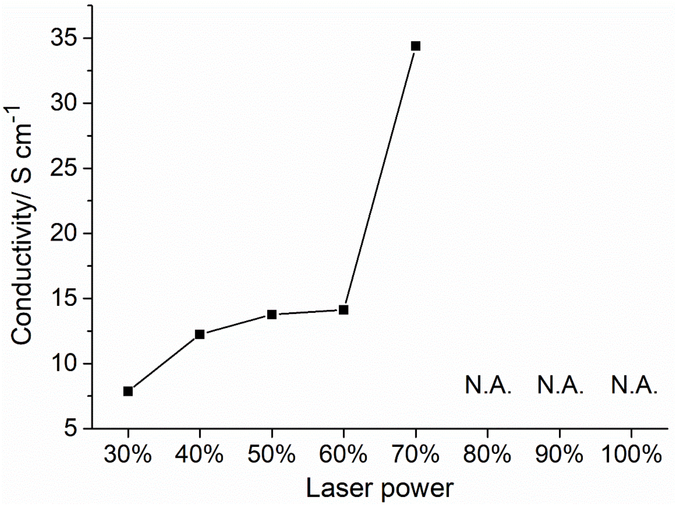

Figure S4. Calculated conductivity (σ) of the resulting LIG30–70% (data for LIG80–100% are not available due to the inability to detect the thickness using the Dektak surface profilometer). The calculation is based on $\sigma = 1/(Rs{*}t)$, in which σ is the conductivity (S cm^{-1}), *Rs* is the sheet resistance (Ω sqr^{-1}) and *t* is the thickness (cm^{-1}).

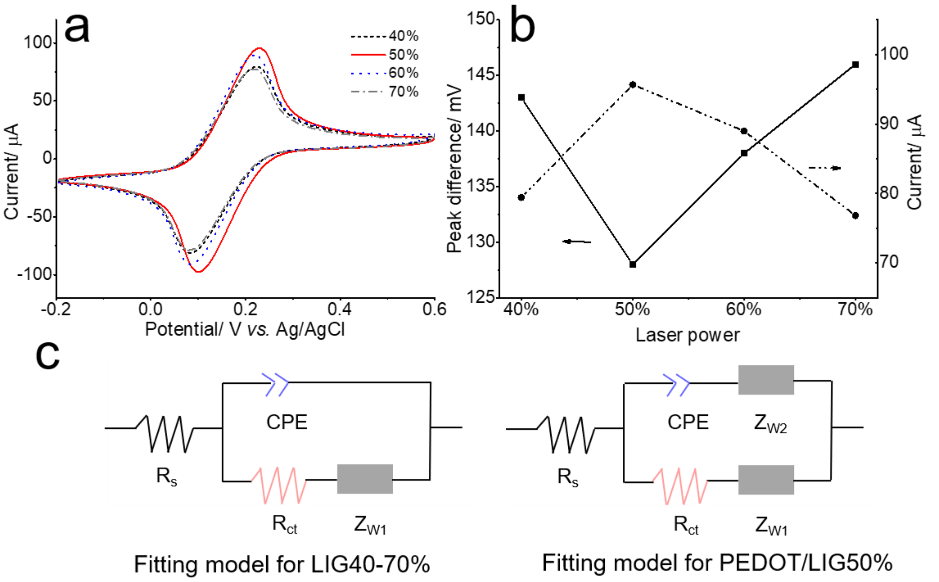

Electrode	Rs (Ω)	Rct (Ω)	Q (μs$^\alpha$/Ω)	α	W1(kΩ/s$^{0.5}$)	W2(kΩ/s$^{0.5}$)
LIG40%	455	510	11.56	0.799	1.421	-
LIG50%	435	325	16.42	0.752	1.754	-
LIG60%	449	468	15.66	0.775	1.482	-
LIG70%	450	527	14.84	0.811	1.149	-
PEDOT/LIG50%	357	21	42.94	0.739	0.670	0.962

Figure S5. (a) Cyclic voltammograms of LIG40–70% working electrode (diameter 3 mm) in 5 mM $Fe(CN)_6^{3-/4-}$ in 0.1 M KCl, scan rate of 50 mV s^{-1}; (b) corresponding redox peak potential difference and anodic peak current of different LIG40–70%; (c) equivalent circuit fitting model and corresponding EIS fitted parameters of LIG40–70% and PEDOT/LIG50% in 5 mM $Fe(CN)_6^{3-/4-}$ in 0.1 M KCl.

The effect of laser power on the electrochemical properties of LIG was investigated by cyclic voltammetry (CV) and electrochemical impedance spectroscopy (EIS), in 5 mM $Fe(CN)_6^{3-/4-}$ in 0.1 M KCl for LIG40–70% due to their relatSive low sheet resistance. Electrochemical measurements of LIG40-70% were performed using a 3-electrode system with an internal Ag/AgCl RE and CE. Figure S5a shows that all the LIG electrodes exhibited quasi-reversible peaks with different peak-to-peak differences (ΔEp) and peak height currents (Ip) as summarized in Figure S5b. The ΔEp decreases from 0.143 V for LIG40% to 0.128 V for LIG50%, which increases up to 0.146 V for LIG70%. And with the increase of laser power, the Ip increases gradually from 79.5 µA (LIG40%) to 95.7 µA (LIG50%) and then decreases to 76.9 µA for LIG70%.

The Nyquist data (Figure S5c) for various LIG electrode in 5 mM $Fe(CN)_6^{3-/4-}$ in 0.1 M KCl was fitted by Randles circuit, including a solution resistance (Rs), a charge-transfer resistance (Rct), a Warburg impedance (W) and a constant phase element (CPE, Q) with a n value (n = 0 is a pure resistor,

n = 1 is a pure capacitor). The Rct decreases from 510 Ω for LIG 40% to 325 Ω for LIG 50%, and increases to 468 and 527 Ω for LIG60% and LIG70%, respectively.

For the EIS results of PEDOT/LIG and LIG (Figure 2c), both of the Nyquist plots consist of a semicircle in the high frequency region related to the charge-transfer limited process and a ~45˚ Warburg diffusion line in the middle-low frequency region corresponding to a semi-infinite diffusion process, which is typical for an electrochemical reaction process at an interface. Additionally, the low frequency region of PEDOT/LIG shifted from ~45˚ to ~90˚, indicating the additional capacitive regimes compared to bare LIG, due to the introduction of the PEDOT nano-film. Therefore, as illustrated in Figure S5c, extra Warburg (Z_{W2}) is added representing the finite-length Warburg diffusion impedance due to diffusion of charge compensating counterions in the polymer film[7-9]. Due to the doping and de-doping process of the PEDOT, exchange of the supporting electrolyte ions (i.e., K^+ and Cl^-) occurs also at the PEDOT|solution interface. As discussed in the main text, the Rct value exhibited an approximately 15-fold decrease from 325 Ω (bare LIG) to 21 Ω (PEDOT/LIG), which reveals the improved electrode kinetics originating from the excellent electronic/ionic conductivity of PEDOT. Besides this, the introduction of PEDOT onto LIG results in an increase of the capacitance to 9.82 µF from 3.22 µF of bare LIG in 5 mM $Fe(CN)_6^{3-/4-}$ in 0.1 M KCl under the OCP of 0.20 V calculated using the following equation[10]:

$$Z_{CPE} = 1 / [(j\ \omega)^{\alpha}\ Q]$$

$$C = (Q\ R)^{(1/n)}/R$$

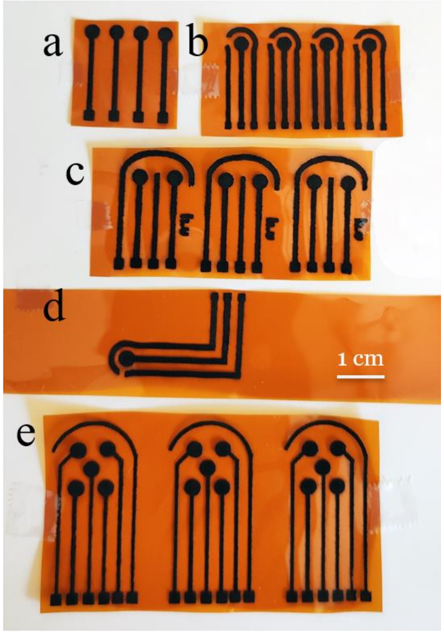

Figure S6. Digital image of (a) patternable standalone working electrodes (3 mm diameter); (b) 3-electrode systems including working electrode (WE), reference electrode (RE) and counter electrode (CE); (c) dual channel electrode system; (d) 3-electrode systems in a 3D wristband design; and (e) a multichannel electrode system.

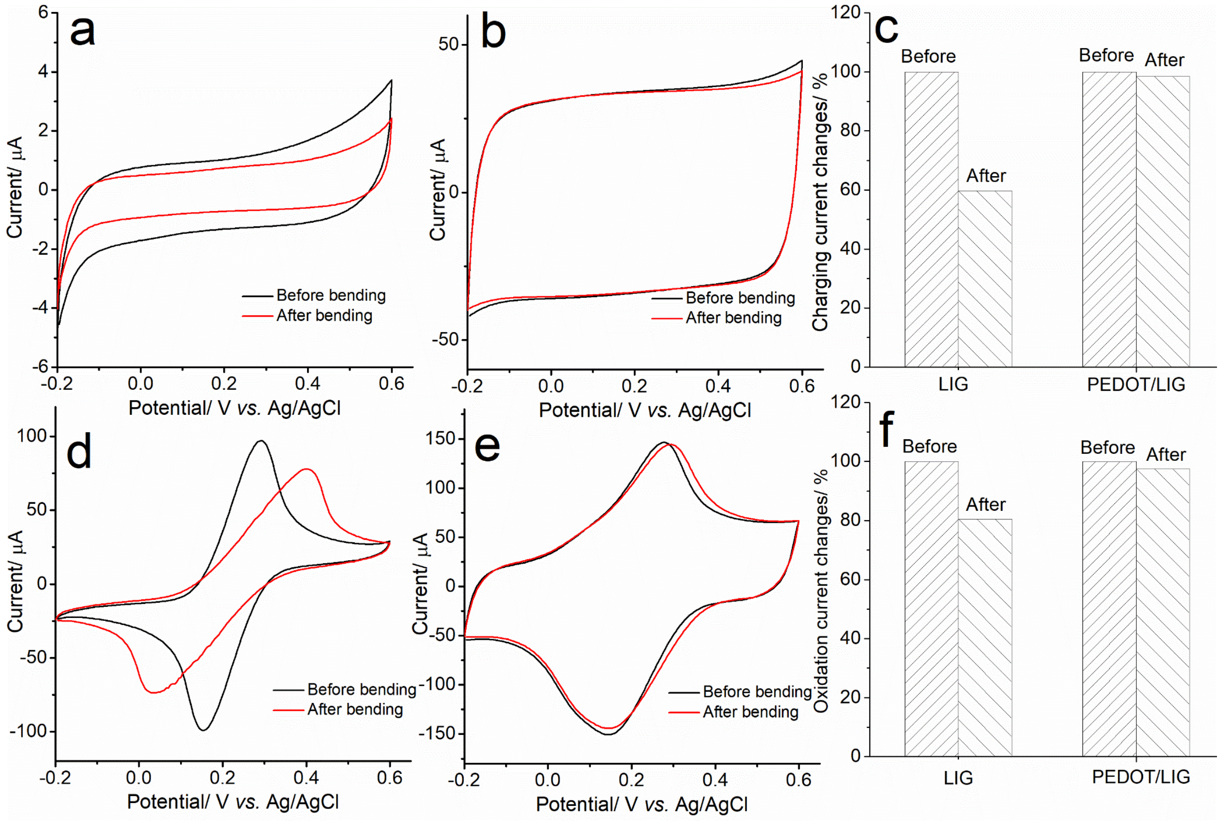

Figure S7. Bending stability test of LIG and PEDOT/LIG standalone working electrodes bent by 120° in two directions 200 times. Cyclic voltammograms of LIG (a) and PEDOT/LIG (b) in 0.1 M KCl before and after bending, scan rate of 50 mV s^{-1}; (c) corresponding charging current changes before and after bending, the charging currents of LIG and PEDOT/LIG before bending were defined as 100%, respectively; cyclic voltammograms of LIG (d) and PEDOT/LIG (e) in 5 mM $Fe(CN)_6^{3-/4-}$ in 0.1 M KCl before and after bending, scan rate of 50 mV s^{-1}; (f) corresponding oxidation current value changes before and after bending, the oxidation current values of LIG and PEDOT/LIG before bending were defined as 100%, respectively.

Figure S7a shows the large change of the background current for LIG electrode after bending, while the CV curve for PEDOT/LIG (Figure S7b) after bending exhibits negligible differences compared to that of before bending. As calculated in Figure S7c, 98.5% of the charging current was remained for PEDOT/LIG electrode after bending, which is much better than that of the bare LIG electrode with only 59.7% remained. The obvious decrease of charging current for bare LIG after bending can be ascribed to the generation of cracks and loss of graphene during bending cycles, which is consistent with Figure 2d and f in the main manuscript. After the nano-deposition of PEDOT, the interconnected graphene networks are reinforced resulting in improved structural stability and thus improved electrochemical stability.

To further evaluate the electrochemical performance of the PEDOT/LIG electrode, CVs of PEDOT/LIG and bare LIG electrodes were recorded in 5 mM $Fe(CN)_6^{3-/4-}$ in 0.1 M KCl to observe the

redox reactions on the electrode surface. As seen in Figure S7d, after bending, the peak-to-peak potential difference (ΔP) for bare LIG becomes much broader (372 mV) compared to before bending (135 mV), while the oxidation current value decreased by 19.9%, indicating that the electrode kinetics were hindered. For PEDOT/LIG (Figure S7e), the ΔP value exhibited no significant difference before (129 mV) and after bending (146 mV) with only a 3.5% decrease of the oxidation current value (Figure S7f).

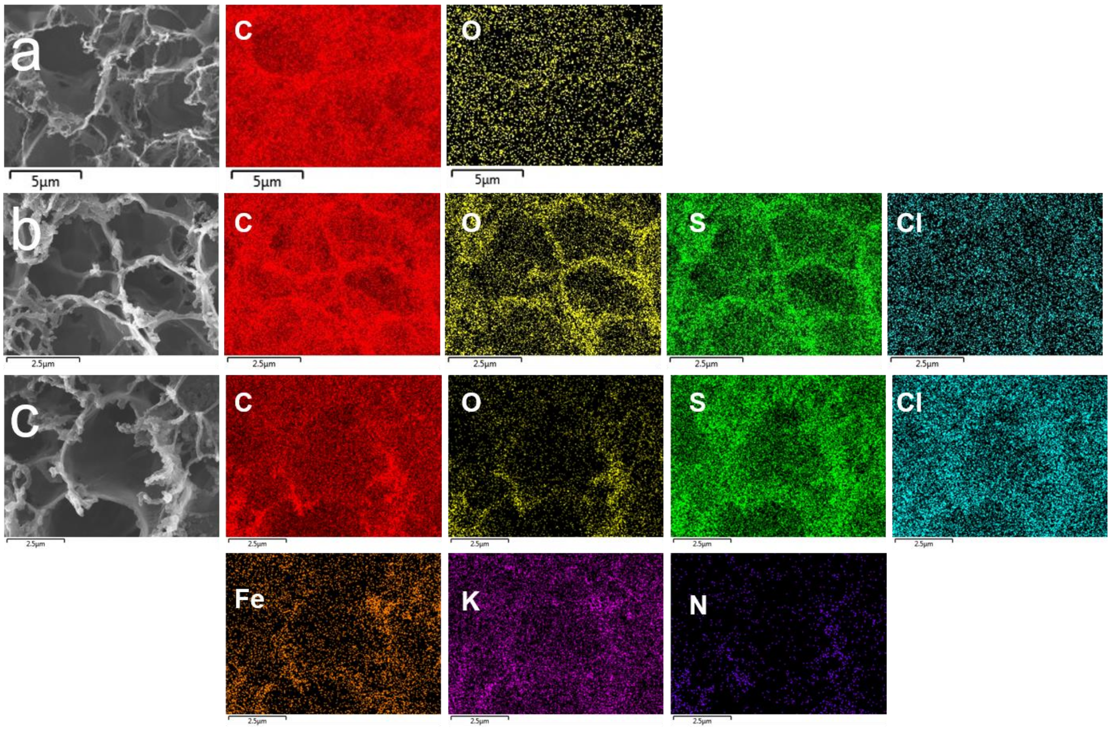

Figure S8. EDS mapping of (a) bare LIG, (b) PEDOT/LIG and (c) PB-PEDOT/LIG.

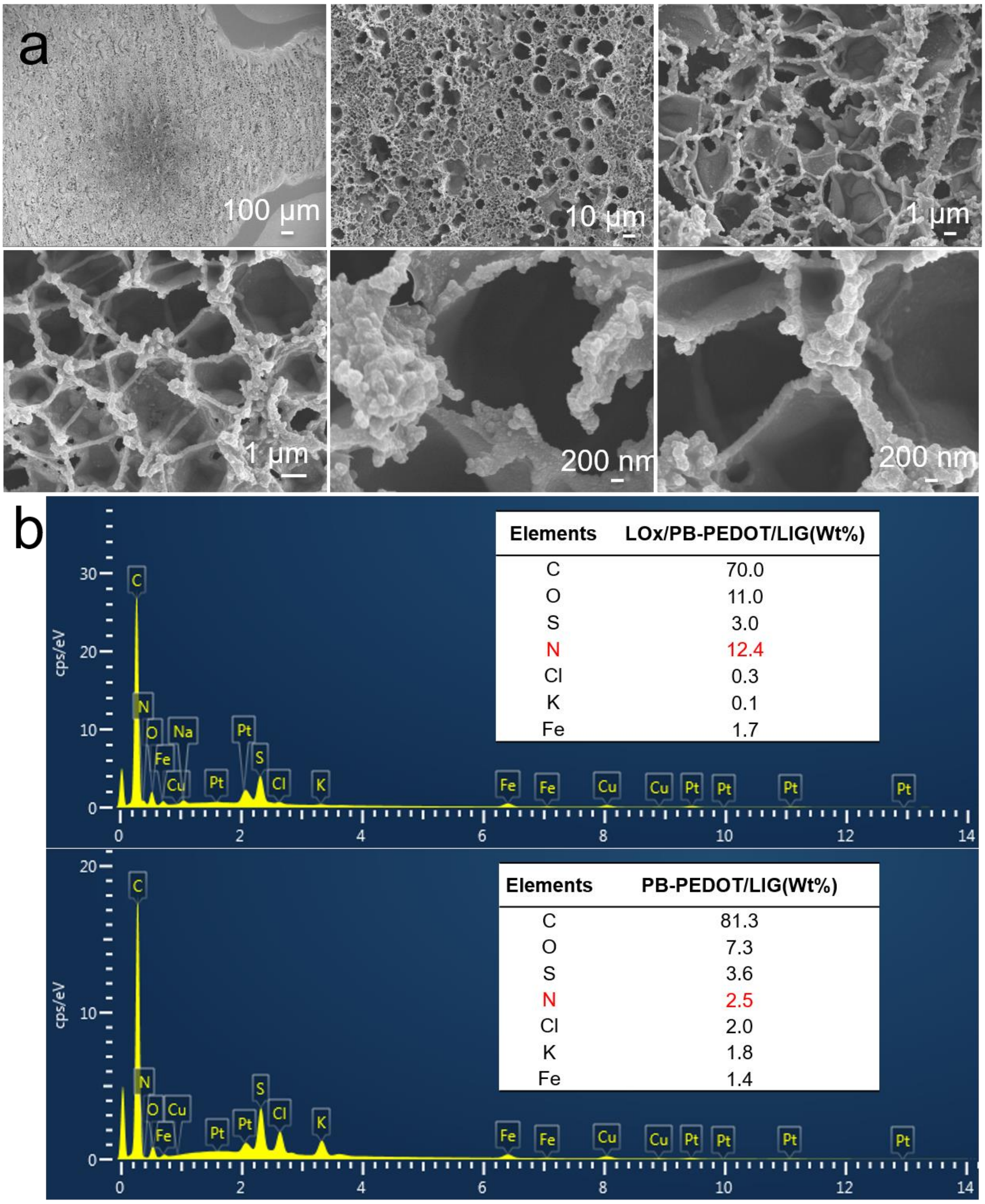

Figure S9. (a) SEM image of LOx/PB-PEDOT/LIG; (b) EDS spectrum of LOx/PB-PEDOT/LIG and PB-PEDOT/LIG.

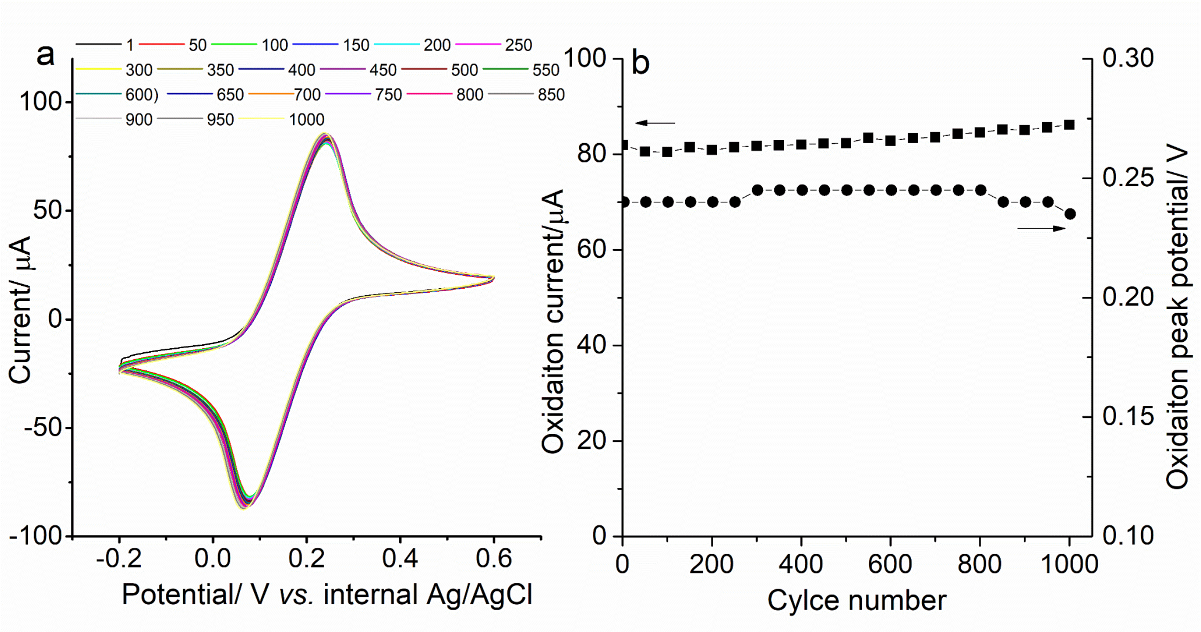

Figure S10. (a) Stability test of Ag/AgCl ink paste as an internal reference electrode with successive 1000 cycles in 5 mM $Fe(CN)_6^{3-}/^{4-}$ in 0.1 M KCl, scan rate of 50 mV s^{-1}; (b) corresponding oxidation current change and oxidation peak potential change upon the bending.

The stability of the Ag/AgCl ink as an internal reference in the integrated 3-electrode system was evaluated using CVs with a $Fe(CN)_6^{3-/4-}$ redox probe for 1000 cycles over the range of -0.2~0.6 V, and the results are shown in Figure S10. No significant decay or deviation of the redox peaks was observed over the 1000 cycles as shown in Figure S10a. Taking the anodic oxidation peak as the example (Figure S10b), the oxidation current demonstrated a slight increase of 5% from 81.91 µA (1st cycle) to 86.20 µA (1000th cycle). In addition, all the oxidation peak potentials for each cycle locate in the range 0.240~0.245 V, with only 5 mV drift. This result indicates the good stability of the internal reference electrode for continuous measurement.

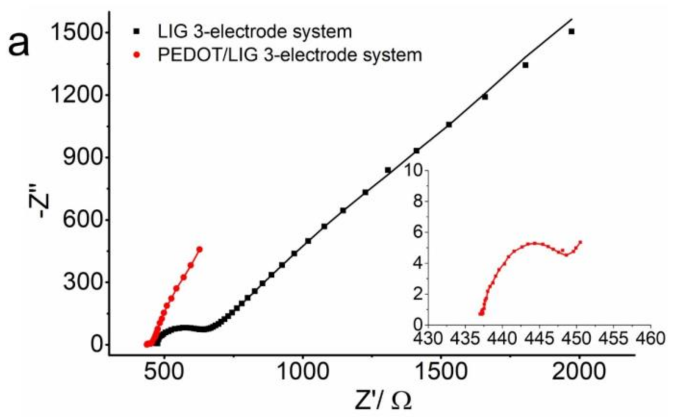

b

Electrode	Rs (Ω)	Rct (Ω)	Q (μs$^{\alpha}$/Ω)	α	W1(kΩ/s$^{0.5}$)	W2(kΩ/s$^{0.5}$)
LIG	465	228	9.76	0.672	2.866	-
PEDOT/LIG	437	11	36.7	0.860	0.479	0.186

Figure S11. (a) Nyquist plots of the integrated 3-electrode system based on PEDOT/LIG (red curve) and bare LIG (black curve) in 5 mM $Fe(CN)_6^{3-/4-}$ in 0.1 M KCl; (b) corresponding EIS fitted parameters.

EIS measurements were performed to evaluate the custom-built integrated 3-electrode system based on PEDOT/LIG and bare LIG. The Nyquist plots (Figure S11a) show a similar characteristics as the LIG and PEDOT/LIG standalone working electrode as Figure 2c. Based on the equivalent circuit fitting model in Figure S5, the corresponding fitting parameters are listed in Figure S11b. The Rct value from the Nyquist plot of PEDOT/LIG 3-electrode system is 11 Ω, which is dramatically smaller than that of the bare LIG 3-electrode system. The good performing EIS also supports the feasibility of the custom-built integrated 3-electrode system for further fabrication of a flexible skin patch lactate biosensor.

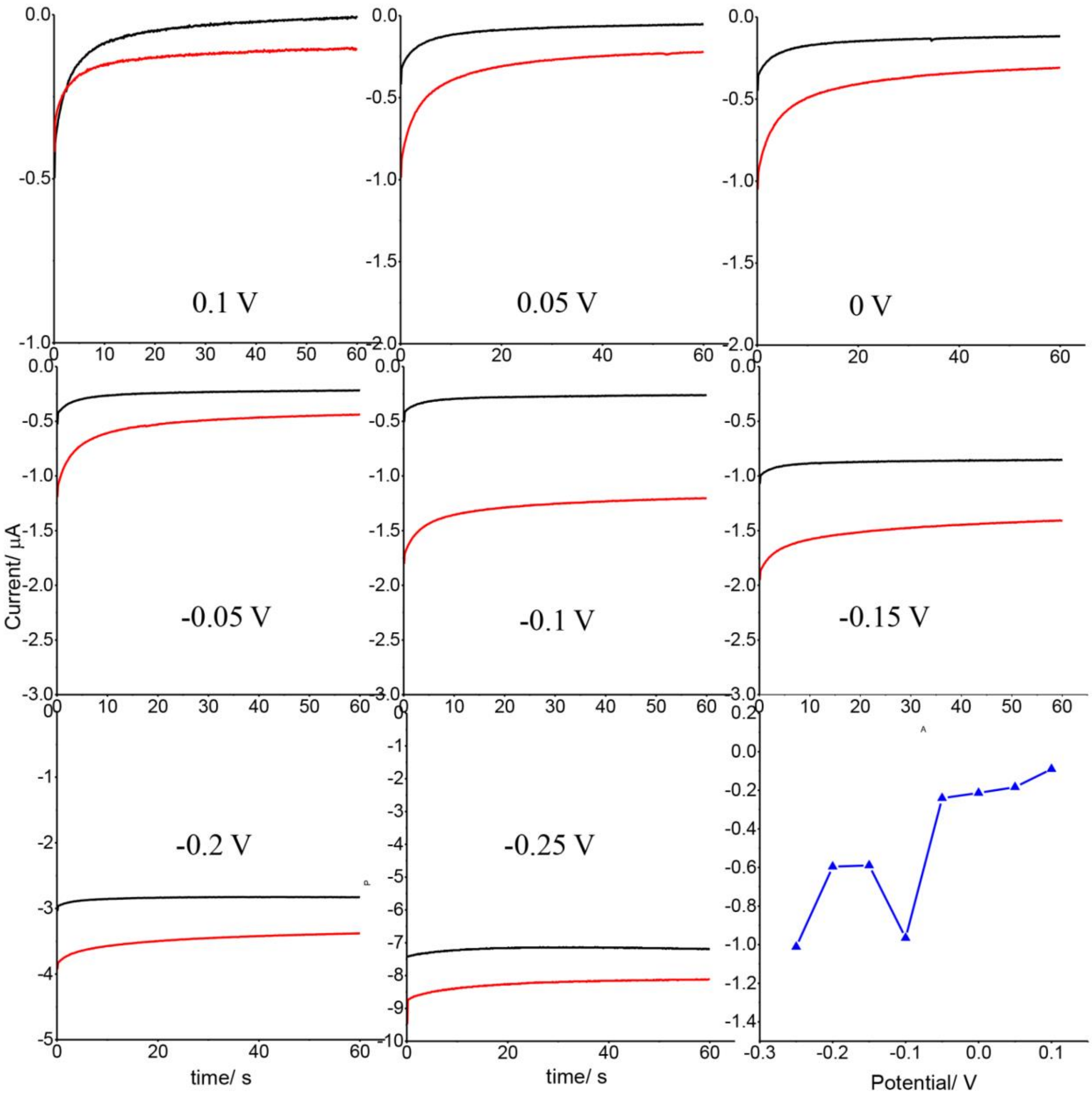

Figure S12. Amperometric response of the assembled 3-electrode system to 0.5 mM lactate in artificial sweat at different potentials ranging from 0.1 to -0.25 V. Black curve: amperometry in blank artificial sweat; red curve: amperometry in 0.5 mM lactate in artificial sweat.

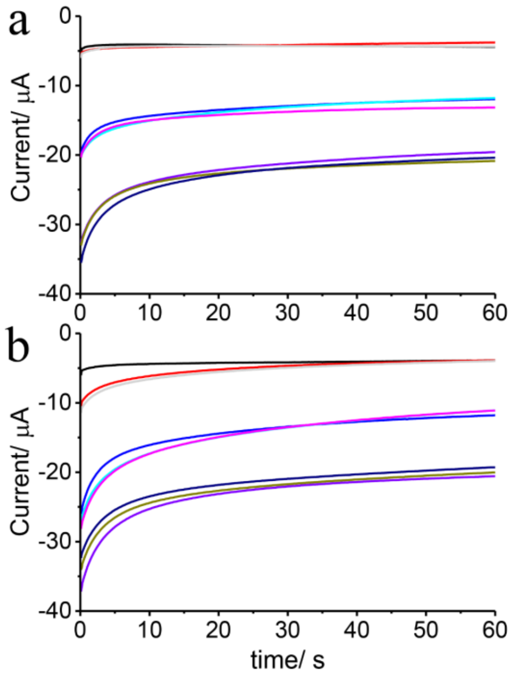

Figure S13. Amperometric response of (a) 2D patch and (b) 3D wristband to 2, 6, 10 mM lactate in artificial sweat at the skin model surface.

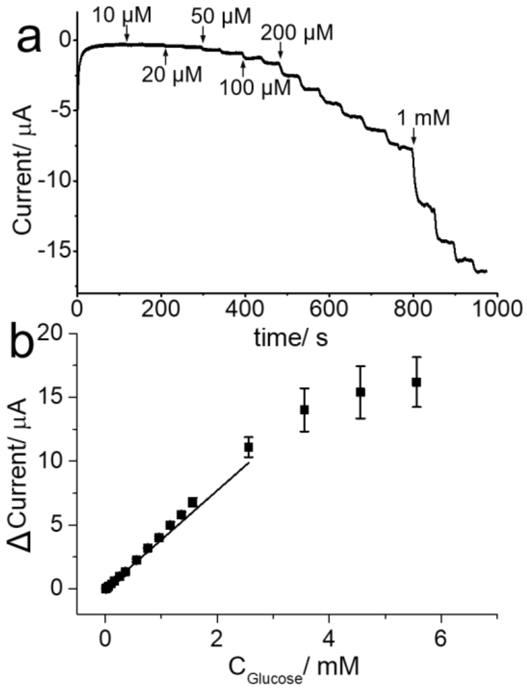

Figure S14. (a) Current-time response curve of GOx encapsulated PB-PEDOT/LIG electrode to successive addition of glucose in 0.1 M PBS (pH=6.4) at 0 V; (b) corresponding calibration curve of GOx encapsulated PB-PEDOT/LIG working electrode to lactate, n=3.

Table S1. Comparison with other PB-based materials for sensing of H_2O_2 and LOx-based electrode for biosensing of lactate described in the literature.

Analyte	Electrode	Potential/ V	Sensitivity/ µA mM^{-1} (µA mM^{-1} cm^{-2})	Linear range/ µM	LOD/ µM	Reference
H_2O_2	PB-PEDOT/LIG	0	24.33 (344.1)	10–1760	2.5	**This work**
	PB NPs	0	(156.5)	0.5–2000	0.1	11
	PEDOT/PB/GCE	0	-	0.5–839	0.16	12
	RGO/PB/GCE	-0.05	51.26	0.8–500	0.25	13
	CB-PB-Nafion/SPCE	-0.05	-	0.2–1000	0.01	14
	PB/PANI/GO/GCE	0	(60.16)	0.5–1275	1.9	15
	PEDOT/PB/Pt	-0.05	37.7	5–1000	1.4	16
	RGO–PB/GCE	-0.1	-	1.6–70	0.5	17
	PBNCs/SnO_2 QDs/RGO/GCE	-0.1	(214)	25-225	0.07	18
Lactate	LOx/PB-PEDOT/LIG	0	11.83 (167.3)	10–1350	6.8	**This work**
	LOx/PB/SPCE	-	(180)	0.5–1000	0.1	19
	LOx/Pt	-	(53)	0-1000	-	20
	LOx/HRP-ferrocene/SPCE	-0.1	(116.9)	1.1-560	0.5	21
	LOx/PtNPs/GCNF/SPCE	0.3	(41.3)	10-2000	6.9	22
	LOx/ PtNp-CNF-PDDA/SPCE	0.5	(36.8)	250-1500	11.1	23
	$FcMe_2$-LPEI/LOx	0.24	(45±6)	0-5000	-	24
	LOx/PB-carbon/Ag/PEN	0	1.9±0.2	0-1000	-	25
	Au microneedle/Au-MWCNTs/pMB/Lox	0.15	(1473)	10-200	2.4	26

Note: NPs-nanoparticles; GCE-glassy carbon electrode; RGO-reduced graphene oxide; CB-carbon black; SPCE-screen printed carbon electrode; PANI-polyaniline; PBNCs-Prussian blue nanocubes; QDs-quantum dots; GCNF-graphitized carbon nanofibers; FcMe2-LPEI- dimethylferrocene-modified linear poly(ethylenimine) (FcMe2-LPEI) hydrogel; PEN- poly(ethylene naphthalate); MWCNTs- multiwalled carbon nanotubes; pMB-poly-methylene blue.

Table S2. Comparison with amperometric lactate biosensors described in the literature.

Electrode	Potential/ V	Sensitivity/ µA mM^{-1} (µA mM^{-1} cm^{-2})	Linear range/ mM	Testing conditions	Ref
Integrated LOx/PB-PEDOT/LIG	-0.1	2.23 (31.54)	0–18	In artificial sweat mounted as a skin patch on pig skin model	This work
Chit/Lox/TTF/carbon fibre	+0.05	0.644	1–20	In 0.1 M PBS (pH 7.0) mounted as a tattoo on a rigid plastic substrate	27
LOx-PPD/Prussian-blue-graphite	0.042	0.553	0.1–1.0	In 0.1 M PBS (pH 7.0) mounted on a mouthguard	28
LOx-PPD/HRP-MCNTs-PEGDGE/graphite	0	0.74	0–25	In 0.1 M PBS (pH 7.0) mounted on eyeglasses	29
Chit-LOx-BSA/Prussian blue conductive carbon	-0.1	0.096	0–28	In 0.1 M PBS (pH 7.0) mounted on eyeglasses	30
Chit-LOx-BSA/ Prussian blue/ graphite	-0.1	N.A.	0-8	In 0.1 M PBS (pH 7.0) mounted on eyeglasses	31
Chit-LOx/ Prussian blue/gold	-0.1	(14.6)[a]<br>(2.5)[b]	0-5<br>10-30	In artificial sweat mounted on textile	32

Note: Chit-Chitosan, TTF- tetrathiafulvalene, PBS- phosphate buffer, PPD- poly-orthophenylenediamine, HRP- horseradish peroxidase, MWCNTs- carboxy-functionalized multiwalled carbon nanotubes, PEGDGE- poly(ethylene glycol) diglycidyl ether, BSA- bovine serum albumin; [a] Sensitivity in µA mM^{-1} cm^{-2} for the linear range of 0-5 mM, [b] Sensitivity in µA mM^{-1} cm^{-2} for the linear range of 10-30 mM.

Reference

(1) Zykwinska, A.; Domagala, W.; Pilawa, B.; Lapkowski, M. Electrochemical Overoxidation of Poly (3, 4-Ethylenedioxythiophene)—Pedot Studied by Means of in Situ Esr Spectroelectrochemistry. *Electrochim. Acta* **2005,** *50* (7-8), 1625-1633.

(2) Ujvári, M.; Gubicza, J.; Kondratiev, V.; Szekeres, K. J.; Láng, G. Morphological Changes in Electrochemically Deposited Poly (3, 4-Ethylenedioxythiophene) Films During Overoxidation. *J. Solid State Electrochem.* **2015,** *19* (4), 1247-1252.

(3) Kamensky, M.; Eliseeva, S.; Láng, G.; Ujvári, M.; Kondratiev, V. Electrochemical Properties of Overoxidized Poly-3, 4-Ethylenedioxythiophene. *Russ. J. Electrochem.* **2018,** *54* (11), 893-901.

(4) Melato, A.; Mendonça, M.; Abrantes, L. Effect of the Electropolymerisation Conditions on the Electrochemical, Morphological and Structural Properties of Pedoth Films. *J. Solid State Electrochem.* **2009,** *13* (3), 417-426.

(5) Meng, L.; Turner, A. P.; Mak, W. C. Modulating Electrode Kinetics for Discrimination of Dopamine by a Pedot: Cooh Interface Doped with Negatively Charged Tricarboxylate. *ACS Appl. Mater. Inter.* **2019,** *11* (37), 34497-34506.

(6) Karyakin, A. A. Prussian Blue and Its Analogues: Electrochemistry and Analytical Applications. *Electroanalysis: An International Journal Devoted to Fundamental and Practical Aspects of Electroanalysis* **2001,** *13* (10), 813-819.

(7) Sundfors, F.; Bobacka, J.; Ivaska, A.; Lewenstam, A. Kinetics of Electron Transfer between Fe (Cn) 63−/4− and Poly (3, 4-Ethylenedioxythiophene) Studied by Electrochemical Impedance Spectroscopy. *Electrochim. Acta* **2002,** *47* (13-14), 2245-2251.

(8) Sundfors, F.; Bobacka, J. Eis Study of the Redox Reaction of Fe (Cn) 63−/4− at Poly (3, 4-Ethylenedioxythiophene) Electrodes: Influence of Dc Potential and Cox: Cred Ratio. *J. Electroanal. Chem.* **2004,** *572* (2), 309-316.

(9) Meng, L.; Turner, A. P.; Mak, W. C. Tunable 3d Nanofibrous and Bio-Functionalised Pedot Network Explored as a Conducting Polymer-Based Biosensor. *Biosens. Bioelectron.* **2020,** *159,* 112181.

(10) Vagin, M. Y.; Sekretaryova, A. N.; Ivanov, I. G.; Håkansson, A.; Iakimov, T.; Syväjärvi, M.; Yakimova, R.; Lundström, I.; Eriksson, M. Monitoring of Epitaxial Graphene Anodization. *Electrochim. Acta* **2017,** *238,* 91-98.

(11) Pandey, P. C.; Panday, D. Tetrahydrofuran and Hydrogen Peroxide Mediated Conversion of Potassium Hexacyanoferrate into Prussian Blue Nanoparticles: Application to Hydrogen Peroxide Sensing. *Electrochim. Acta* **2016,** *190,* 758-765.

(12) Wang, J.; Wang, Y.; Cui, M.; Xu, S.; Luo, X. Enzymeless Voltammetric Hydrogen Peroxide Sensor Based on the Use of Pedot Doped with Prussian Blue Nanoparticles. *Microchim. Acta* **2017,** *184* (2), 483-489.

(13) Li, J.; Jiang, Y.; Zhai, Y.; Liu, H.; Li, L. Prussian Blue/Reduced Graphene Oxide Composite for the Amperometric Determination of Dopamine and Hydrogen Peroxide. *Anal. Lett.* **2015,** *48* (17), 2786-2798.
(14) Rojas, D.; Della Pelle, F.; Del Carlo, M.; d'Angelo, M.; Dominguez-Benot, R.; Cimini, A.; Escarpa, A.; Compagnone, D. Electrodeposited Prussian Blue on Carbon Black Modified Disposable Electrodes for Direct Enzyme-Free H2o2 Sensing in a Parkinson's Disease in Vitro Model. *Sensor. Actuat. B-Chem* **2018,** *275*, 402-408.
(15) Yang, Z.; Zheng, X.; Zheng, J. A Facile One-Step Synthesis of Prussian Blue/Polyaniline/Graphene Oxide Nanocomposites for Electrochemical Sensing of Hydrogen Peroxide. *Synth. Met.* **2016,** *221*, 153-158.
(16) Lete, C.; Marin, M.; Anghel, E. M.; Preda, L.; Matei, C.; Lupu, S. Sinusoidal Voltage Electrodeposition of Pedot-Prussian Blue Nanoparticles Composite and Its Application to Amperometric Sensing of H2o2 in Human Blood. *Materials Science and Engineering: C* **2019,** *102*, 661-669.
(17) Zhang, M.; Hou, C.; Halder, A.; Ulstrup, J.; Chi, Q. Interlocked Graphene–Prussian Blue Hybrid Composites Enable Multifunctional Electrochemical Applications. *Biosens. Bioelectron.* **2017,** *89*, 570-577.
(18) Chauhan, S.; Sahoo, S.; Satpati, A. K.; Sahoo, P. K. Prussian Blue Nanocubes‐ Sno2 Quantum Dots‐ Reduced Graphene Oxide Ternary Nanocomposite: An Efficient Non‐ Noble‐ Metal Electrocatalyst for Non‐ Enzymatic Detection of H2o2. *Electroanalysis*.
(19) Yashina, E. I.; Borisova, A. V.; Karyakina, E. E.; Shchegolikhina, O. I.; Vagin, M. Y.; Sakharov, D. A.; Tonevitsky, A. G.; Karyakin, A. A. Sol− Gel Immobilization of Lactate Oxidase from Organic Solvent: Toward the Advanced Lactate Biosensor. *Anal. Chem.* **2010,** *82* (5), 1601-1604.
(20) Thomas, N.; Lähdesmäki, I.; Parviz, B. A. A Contact Lens with an Integrated Lactate Sensor. *Sensor. Actuat. B-Chem* **2012,** *162* (1), 128-134.
(21) Pérez, S.; Fàbregas, E. Amperometric Bienzymatic Biosensor for L-Lactate Analysis in Wine and Beer Samples. *Analyst* **2012,** *137* (16), 3854-3861.
(22) Loaiza, O. A.; Lamas-Ardisana, P. J.; Añorga, L.; Jubete, E.; Ruiz, V.; Borghei, M.; Cabañero, G.; Grande, H. J. Graphitized Carbon Nanofiber–Pt Nanoparticle Hybrids as Sensitive Tool for Preparation of Screen Printing Biosensors. Detection of Lactate in Wines and Ciders. *Bioelectrochemistry* **2015,** *101*, 58-65.
(23) Lamas-Ardisana, P. J.; Loaiza, O. A.; Añorga, L.; Jubete, E.; Borghei, M.; Ruiz, V.; Ochoteco, E.; Cabañero, G.; Grande, H. J. Disposable Amperometric Biosensor Based on Lactate Oxidase Immobilised on Platinum Nanoparticle-Decorated Carbon Nanofiber and Poly (Diallyldimethylammonium Chloride) Films. *Biosens. Bioelectron.* **2014,** *56*, 345-351.

(24) Hickey, D. P.; Reid, R. C.; Milton, R. D.; Minteer, S. D. A Self-Powered Amperometric Lactate Biosensor Based on Lactate Oxidase Immobilized in Dimethylferrocene-Modified Lpei. *Biosens. Bioelectron.* **2016,** *77*, 26-31.

(25) Shiwaku, R.; Matsui, H.; Nagamine, K.; Uematsu, M.; Mano, T.; Maruyama, Y.; Nomura, A.; Tsuchiya, K.; Hayasaka, K.; Takeda, Y. A Printed Organic Amplification System for Wearable Potentiometric Electrochemical Sensors. *Scientific reports* **2018,** *8* (1), 1-8.

(26) Bollella, P.; Sharma, S.; Cass, A. E. G.; Antiochia, R. Microneedle-Based Biosensor for Minimally-Invasive Lactate Detection. *Biosens. Bioelectron.* **2019,** *123*, 152-159.

(27) Jia, W.; Bandodkar, A. J.; Valdés-Ramírez, G.; Windmiller, J. R.; Yang, Z.; Ramírez, J.; Chan, G.; Wang, J. Electrochemical Tattoo Biosensors for Real-Time Noninvasive Lactate Monitoring in Human Perspiration. *Anal. Chem.* **2013,** *85* (14), 6553-6560.

(28) Kim, J.; Valdés-Ramírez, G.; Bandodkar, A. J.; Jia, W.; Martinez, A. G.; Ramírez, J.; Mercier, P.; Wang, J. Non-Invasive Mouthguard Biosensor for Continuous Salivary Monitoring of Metabolites. *Analyst* **2014,** *139* (7), 1632-1636.

(29) Zhang, L.; Liu, J.; Fu, Z.; Qi, L. A Wearable Biosensor Based on Bienzyme Gel-Membrane for Sweat Lactate Monitoring by Mounting on Eyeglasses. *Journal of nanoscience and nanotechnology* **2020,** *20* (3), 1495-1503.

(30) Imani, S.; Bandodkar, A. J.; Mohan, A. V.; Kumar, R.; Yu, S.; Wang, J.; Mercier, P. P. A Wearable Chemical–Electrophysiological Hybrid Biosensing System for Real-Time Health and Fitness Monitoring. *Nat. Commun.* **2016,** *7* (1), 1-7.

(31) Sempionatto, J. R.; Nakagawa, T.; Pavinatto, A.; Mensah, S. T.; Imani, S.; Mercier, P.; Wang, J. Eyeglasses Based Wireless Electrolyte and Metabolite Sensor Platform. *Lab Chip* **2017,** *17* (10), 1834-1842.

(32) Wang, R.; Zhai, Q.; An, T.; Gong, S.; Cheng, W. Stretchable Gold Fiber-Based Wearable Textile Electrochemical Biosensor for Lactate Monitoring in Sweat. *Talanta* **2021,** *222*, 121484.
